# Polysaccharide biosynthetic pathway profiling and putative gene mining of *Dendrobium moniliforme* using RNA-Seq in different tissues

**DOI:** 10.1186/s12870-019-2138-7

**Published:** 2019-11-27

**Authors:** Yingdan Yuan, Jinchi Zhang, Justin Kallman, Xin Liu, Miaojing Meng, Jie Lin

**Affiliations:** 1 Co-Innovation Center for Sustainable Forestry in Southern China, Nanjing Forestry University, Nanjing, 210037 China; 2 Jiangsu Province Key Laboratory of Soil and Water Conservation and Ecological Restoration, Nanjing Forestry University, Nanjing, 210037 China; 3 University of Miami, Coral Gables, 33146 USA

**Keywords:** *Dendrobium moniliforme*, Transcriptome, Polysaccharides synthesis, Glycosyltransferase

## Abstract

**Background:**

*Dendrobium moniliforme* (Linnaeus) Swartz is a well-known plant used in traditional Chinese medicine due to bioactive constituents. Polysaccharides are the main medicinal ingredients, yet no studies have been published on polysaccharide biosynthesis in *D. moniliforme*. To comprehensively investigate the polysaccharide at the transcription level, we performed de novo transcriptome sequencing for the first time to produce a comprehensive transcriptome of *D. moniliforme*.

**Results:**

In our study, a database of 562,580 unigenes (average length = 1115.67 bases) was generated by performing transcriptome sequencing. Based on the gene annotation of the transcriptome, we identified 1204 carbohydrate-active related unigenes against CAZy database, including 417 glycosyltransferase genes (GTs), 780 glycoside hydrolases (GHs), 19 carbohydrate esterases (CEs), 75 carbohydrate-binding modules (CBMs), and 44 polysaccharide lyases (PLs). In the cellulose synthase family, 21 differential expression genes (DEGs) related to polysaccharide were identified. Subsequently, the tissue-specific expression patterns of the genes involved in polysaccharide pathway were investigated, which provide understanding of the biosynthesis and regulation of DMP at the molecular level. The two key enzyme genes (Susy and SPS) involved in the polysaccharide pathway were identified, and their expression patterns in different tissues were further analyzed using quantitative real-time PCR.

**Conclusions:**

We determined the content of polysaccharides from *Dendrobium moniliforme* under different tissues, and we obtained a large number of differential genes by transcriptome sequencing. This database provides a pool of candidate genes involved in biosynthesis of polysaccharides in *D. moniliforme*. Furthermore, the comprehensive analysis and characterization of the significant pathways are expected to give a better insight regarding the diversity of chemical composition, synthetic characteristics, and the regulatory mechanism which operate in this medical herb.

## Background

*Dendrobium moniliforme* (Linnaeus) Swartz, is one of the most widespread species in the *Orchidaceae* family, which is a widely cultivated medicinal crop around Asia [1]. *D. moniliforme* has the characteristics of a thin stem, small flower, and it is a representative plant of Chinese traditional medicine that is often processed into finished medicinal materials. It is widely used as folk medicine for antipyretic purpose, benefits to eyes, and as a tonic [2]. *D. moniliforme* plants’ main active ingredients are polysaccharides, alkaloids, bibenzyls, fluorenone compounds, glycosides, amino acids and several trace mineral elements [3]. The stems of *D. moniliforme* are the main medicinal parts, which contain thick water-soluble polysaccharides. Polysaccharide hydrolyzate contains not only mannose and glucose, but also trace amounts of arabinose and xylose [4]. Polysaccharides have been successfully extracted from 100 kinds of plants and are commonly used in the research and development in medicine and health food. They have pharmacological effects of regulating immunity, and acting against tumors and oxidants. Polysaccharides of different origins exhibit different biological activities [5–7].

Polysaccharides are natural macromolecules consisting of multiple monosaccharides units. They represent a structurally diverse class of macromolecules that are widely distributed in nature and play an important role in controlling cell division, regulating cell growth and maintaining normal metabolism of living organisms. Polysaccharides of higher plants are a potential source of pharmacologically active compounds. Numerous studies have shown that polysaccharides isolated from medicinal plants could affect the immune responses both in vivo and in vitro and have the potential of being immunomodulators [8]. The polysaccharide of *Dendrobium* in most of the literature refers to the water-soluble polysaccharide [9, 10]. At present, many people have studied its structure. For the currently separated polysaccharide components of *Dendrobium*, the monosaccharide composition consists mainly of glucose and mannose, and also contains arabinose, galacturonic acid, xylose, rhamnose, galactose and so on [11–13]. All in all, mannose and glucose are the main monosaccharides in these *Dendrobium* species.

The purpose of our research on medicinal ingredients is to be able to apply the extracts of these medicinal ingredients to clinical practice. In order to meet the growing demand for drugs, researchers have gradually applied genetic engineering methods to produce these medicinal components. Therefore, the biosynthesis and metabolism pathways of various active ingredients and the excavation of related key enzyme genes have become important goals of the research on medicinal plants. Functional genomics methods, especially transcriptomics, have shown important applications in discovering the key enzyme genes involved in biosynthesis of secondary metabolites in medicinal plants and elucidating secondary metabolic pathways and regulatory mechanisms. The transcriptome is based on the predecessor (mRNA) of protein in live cells, which represents the real-time situation of life activity and is a very effective method and means to reveal the molecular mechanism of biological growth and physiological activities [14, 15]. In medicinal plant research, transcriptome sequencing technology is widely used in the discovery of new genes [16], metabolic pathways [17], molecular marker mining [18], transcription mapping [19] and so on. A large number of medicinal plants have been studied by RNA-seq transcriptomics, such as *Lycium chinense* [20], *Salvia miltiorrhiza* [21], *Catharanthus roseus* [22], and American ginseng [23]. However, for the first time, we sequenced the transcriptome of three tissues to reveal the polysaccharide synthesis genes and pathways in *D. moniliforme*.

Until now, several transcriptomes in *D. officinale* [24–26] and a few whole genomes have been sequenced [27, 28]. In *Dendrobium* species, a lot of key enzyme-encoding genes involved in the synthesis and metabolic pathways have been identified. However, *Dendrobium* molecular biology research information is still very limited. The molecular marker development and utilization of key genes and other aspects of this study are still in their infancy. The molecular mechanisms underlying polysaccharide biosynthesis and the related metabolic pathways for *D. moniliforme* remain unknown. In this study, we constructed nine transcriptome libraries for root, stem and leaf material in *D. moniliforme*. A total of 1335 glycosyltransferase genes (GTs) and 35 cellulose synthase genes (CesA) were identified, and we also analyzed differentially expressed genes (DEGs) between them. Through the analysis of sequencing data, we understand the biosynthesis of secondary products, particularly in identifying candidate genes involved in DMP biosynthesis. Meanwhile, we verified the quality of our dataset and confirmed these putative DMP genes by quantitative real-time RT-PCR (qRT-PCR).

## Results

### Determination of polysaccharides in three different tissues

**Fig. 1:**
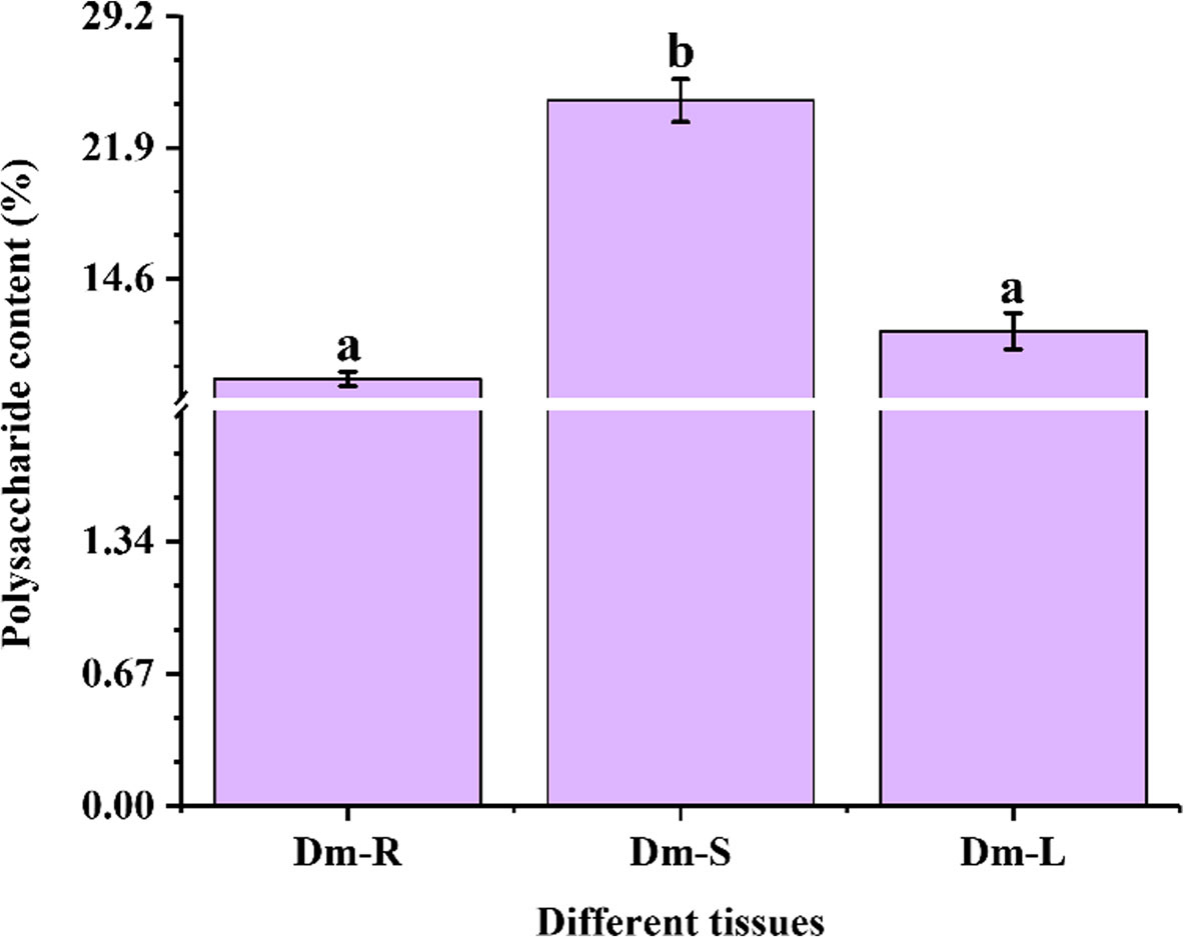
Determination of polysaccharide contents in different tissues of *D. moniliforme.* The a and b letters indicate statistical differences in the results of analysis of variance between different tissues, with a significant difference of *p* < 0.05

Polysaccharide was determined in three different tissues, including leaf, stem and root material. The stem of D. *moniliforme* is its main medicinal part and polysaccharide was mainly concentrated in stems. The highest content of polysaccharide was 24.53% for stems (Fig. 1). From one-way analysis of variance, it is shown that the polysaccharide content of the stem of *D. moniliforme* was significantly different from that of roots and leaves.

### Illumina sequencing and de novo assembly

In this study, nine cDNA libraries were constructed from the three tissues of *D. moniliforme* in three replicates. These libraries were named as follows: root cDNA libraries: Dm_R1, Dm_R2, Dm_R3; stem cDNA libraries: Dm_S1, Dm_S2, Dm_S3; leaf cDNA libraries: Dm_L1, Dm_L2, Dm_L3. In total, 811,451,164 clean reads with a total of 119.89 Gb (Gigabase) were obtained from all of the samples. The base average error rate was 0.01% and the average GC content was 46.36%. The average values of Q20 and Q30 were 98.14 and 94.57%, respectively (Table 1).

**Table 1:** Summary of sequencing quality

Sample ID	Raw reads	Clean reads	Clean bases	Error (%)	Q20 (%)	Q30 (%)	GC (%)
Dm_R1	104,974,434	103,116,856	15.24Gb	0.0126	98.12	94.61	46.41
Dm_R2	112,417,410	110,298,744	16.38Gb	0.012	98.43	95.25	46.63
Dm_R3	93,916,312	91,971,512	13.59Gb	0.0125	98.22	94.7	46.35
Dm_S1	95,188,100	93,364,948	13.79Gb	0.0129	97.99	94.31	46.49
Dm_S2	83,861,418	81,881,046	12.08Gb	0.0127	98.12	94.44	46.1
Dm_S3	95,368,208	92,929,138	13.69Gb	0.0128	98.05	94.27	46.07
Dm_L1	84,551,998	83,097,662	12.29Gb	0.0126	98.13	94.64	46.66
Dm_L2	77,755,976	75,885,672	11.18Gb	0.0127	98.11	94.43	46.36
Dm_L3	80,621,980	78,905,586	11.64Gb	0.0127	98.12	94.45	46.15
Total	828,655,836	811,451,164	119.89Gb				

(1) Q20: percentage of bases with a Phred value > 20; Q30: percentage of bases with a Phred value > 30. (2) Error (%): Base error rate. (3) GC (%): percentage of bases G and C number in the total number of bases

We combined all clean reads to form the *D. moniliforme* transcriptome database by using Trinity software. 679,321 transcripts were identified with the average transcript being 703.77 base pairs (bp). The length distribution of transcripts and unigenes is shown in Additional file 1: Figure S1. Sequencing length of transcripts and unigenes ranged from 201 bp to 16,515 bp. Assembly of clean reads resulted in 562,480 unigenes with a mean length of 567.79 bp. The N50 length of transcripts and unigenes were 1177 bp and 830 bp, respectively.

### Functional annotation, GO, KOG and KEGG classification

A total of 562,480 unigenes were annotated against the Nr, KEGG, Swiss-Prot, Pfam and String databases using the BLAST algorithm (E-value<1E-5) with 14,709 (4.25%) in all five databases (Additional file 1: Figure S2). Analyses showed that 169,702 unigenes (48.99%) had significant matches in the Nr database, 110,415 unigenes (31.87%) in the Swiss-Prot database and 104,684 unigenes (30.22%) in the KEGG database. The lowest proportion occurred in the String database (37,245, 10.75%) and the Pfam was 65,595 (18.94%) annotated unigenes.

**Fig. 2:**
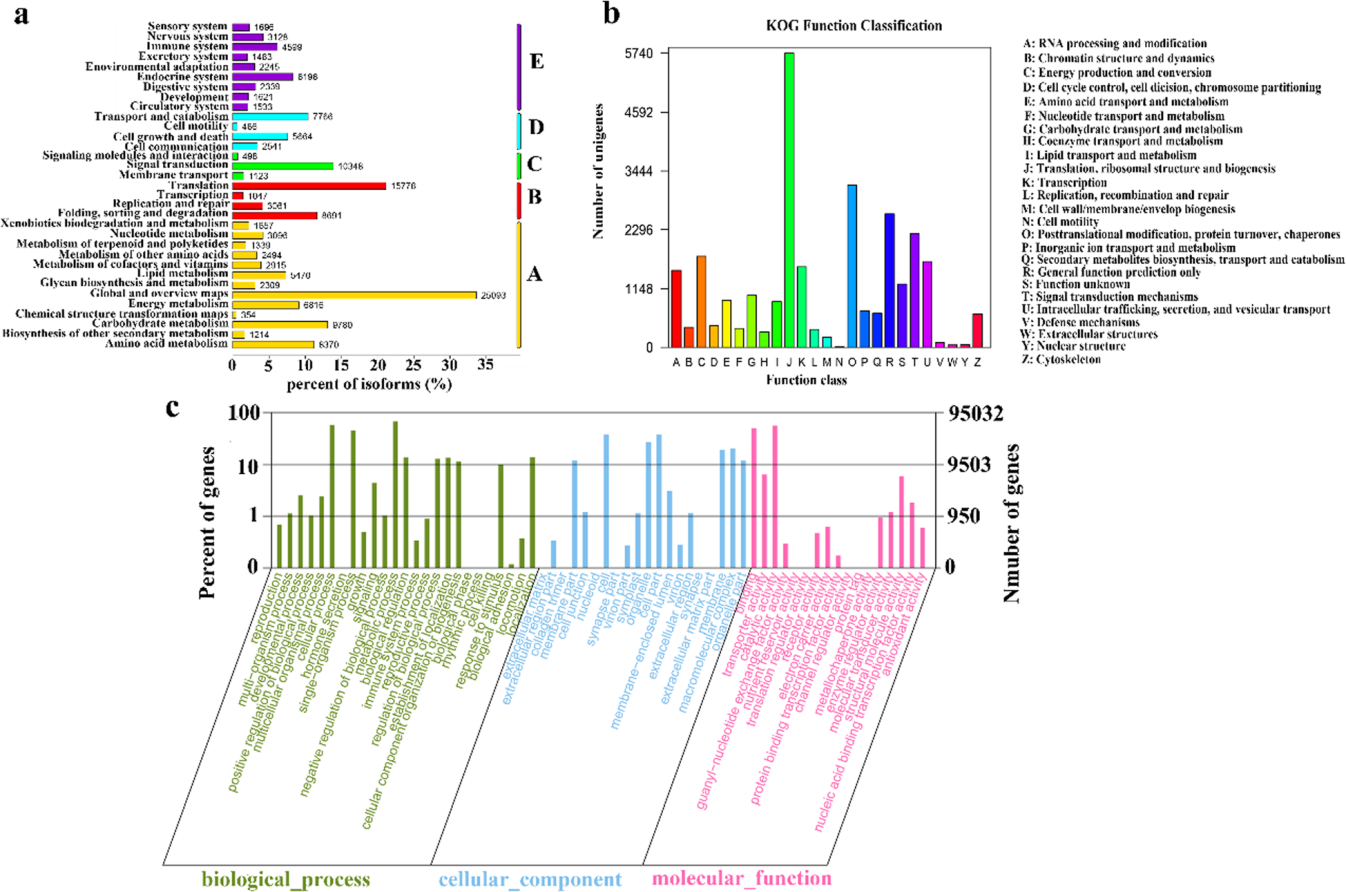
Classification of unigenes and metabolic pathways. **a** KEGG classification of metabolic pathways. The x-axis indicates the percentage of the number of genes annotated to the pathway out of the total number of genes annotated. The y-axis indicates the name of the KEGG metabolic pathway. The genes were divided into five branches according to the KEGG metabolic pathway: Metabolism (A), Genetic Information Processing (B), Environmental Information Processing (C), Cellular Processes (D), Organismal Systems (E). **b** KOG classification of unigenes. The x-axis indicates the name of the 25 groups of KOG. The y-axis indicates the percentage of the number of genes annotated to the group out of the total number of genes annotated. **c** KOG classification of unigenes. The x-axis indicates the name of the 25 groups of KOG. The y-axis indicates the percentage of the number of genes annotated to the group out of the total number of genes annotated

In the KEGG database, a total of 104,684 unigenes were annotated to 33 pathways (Fig. 2a). All unigenes were divided into five categories: Metabolism (A), Genetic Information Processing (B), Environmental Information Processing (C), Cellular Processes (D), Organismal Systems (E). The pathways involving the highest number of unigenes were “global and overview maps” (25,093), followed by “translation” (15,778) and “signal transduction” (10,348).

In addition, the annotated sequences were subjected to the clusters of orthologous groups (KOG) database for functional prediction and classification. There were 37,245 unigenes assigned to KOG classification and divided into 25 specific categories involved in cellular process, signal transduction, metabolism and other processes (Fig. 2b). The three top terms were (J) translation, ribosomal structure and biogenesis, (O) posttranslational modification, protein turnover, chaperones and (R) general function prediction only.

For GO analysis, a total of 95,032 annotated unigenes were classified into three main ontologies and 62 subcategories by using Blast2Go (Fig. 2c). For the biological process (BP) category, genes involved in “metabolic process” (63,996) were high represented. Furthermore, cellular component (CC) categories mainly comprised proteins involved in “cell” (36,019) and “cell part” (36,018). In addition, highly represented in molecular function (MF) categories were “catalytic activity” (52,948) and “binding” (47,656).

### Identification and cluster analysis of differentially expressed genes (DEGs)

**Fig. 3:**
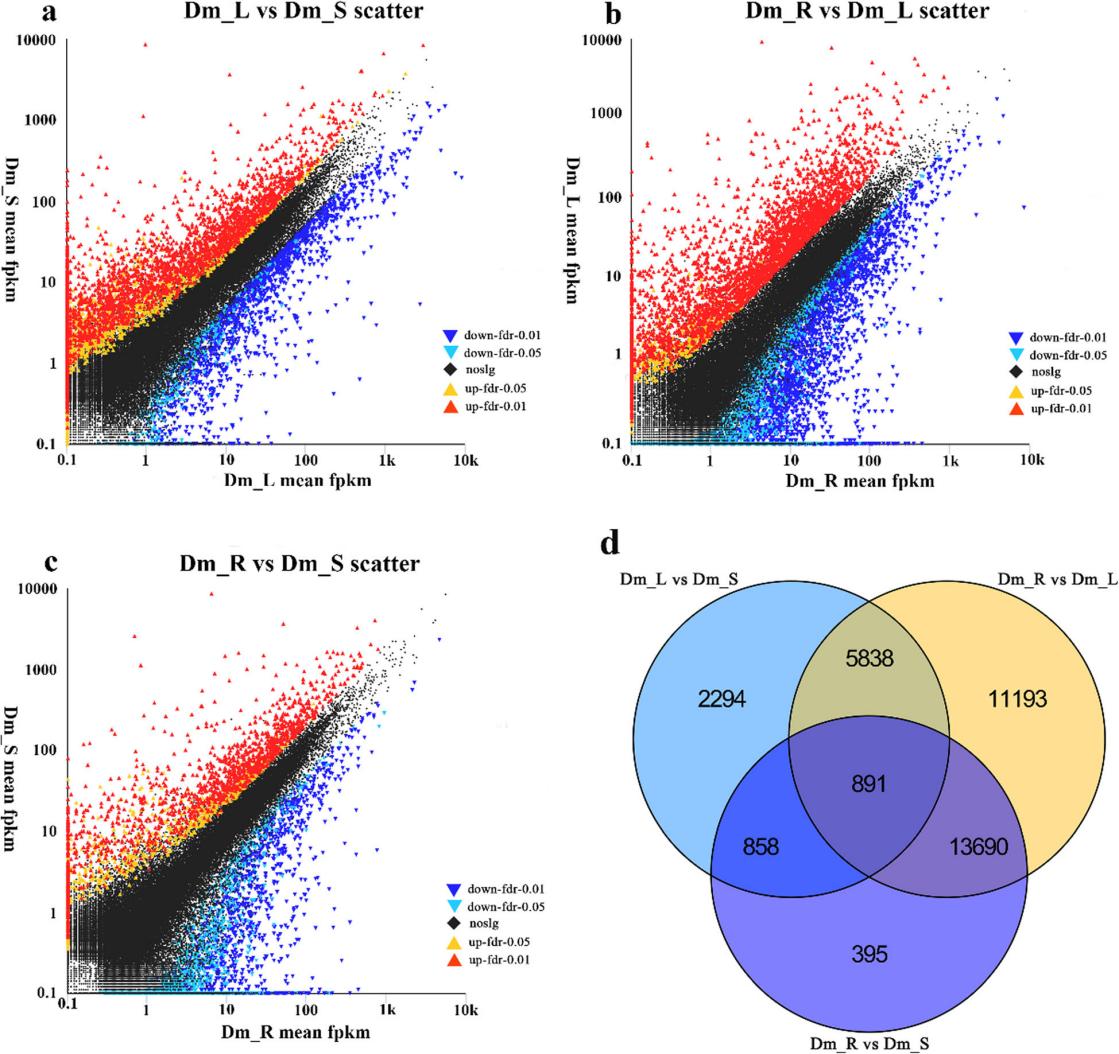
**a**-**c** Scatter plots of the DEGs in different comparisons. The red dots mean significantly up-regulated genes and the blue dots represent significantly down-regulated genes. The black dots represent non-DEGs. **a** Dm_L vs. Dm_S scatter; **b** Dm_R vs. Dm_L scatter; **c** Dm_R vs. Dm_S scatter. **d** Venn diagram of differentially expressed genes (DEGs) in different comparisons. All DEGs are clustered into three comparison groups represented by three ellipses. The overlapping parts of different ellipses represent the number of DEGs in common from those comparison groups

In this study, we examined differential expression of replicated count data by using edgeR. We set “FDR < 0.05& |log_2_FC| ≥ 1” as the standard for significant differences in the expression of genes. When the log_2_FC > 1, the DEG was considered as up-regulated. On the contrary, for log_2_FC < − 1, it was considered as down-regulated. To study gene expression among different tissues, we identified 9881 DEGs between Dm_L and Dm_S, including 7788 up-regulated and 2093 down-regulated DEGs. Comparing Dm_R with Dm_L, the largest number of DEGs were identified. Of these DEGs, 277,625 were down-regulated and 3987 were up-regulated. Finally, there were 15,834 DEGs between Dm_R and Dm_S, 2389 of which were up-regulated and 13,445 of which were down-regulated (Fig. 3d). Furthermore, 2294 and 395 DEGs were expressed uniquely in “Dm_L vs. Dm_S” and “Dm_R vs. Dm_S”, respectively. Also, 11,193 DEGs were expressed uniquely between Dm_R and Dm_L. Among these libraries, 891 DEGs were identified in common (Fig. 3a-c). All the details of DEGs are shown in the Additional files 2, 3 and 4.

**Fig. 4:**
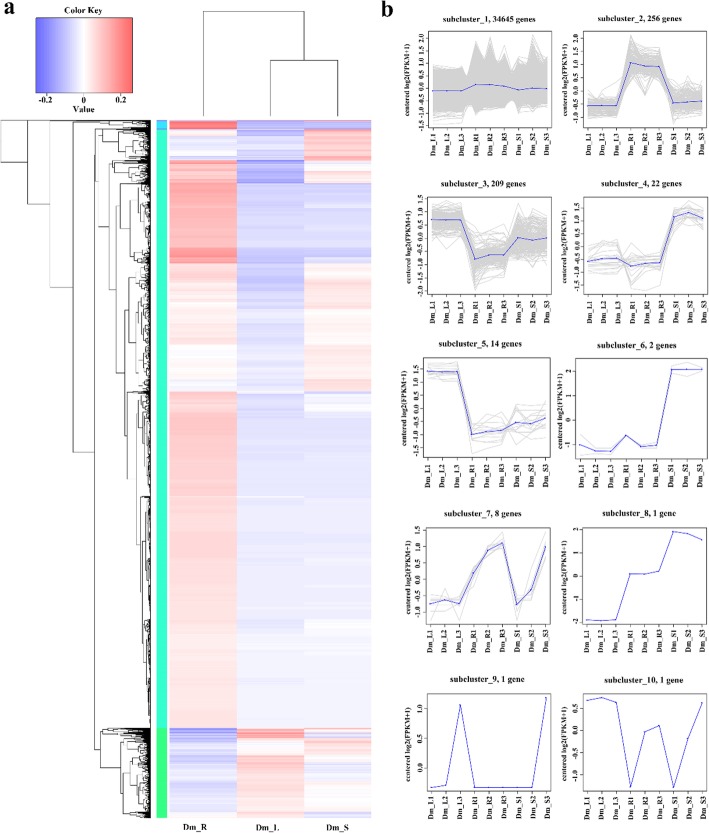
Expression profiles of all DEGs. **a** Heat map of DEGs by hierarchical cluster analysis and FPKM distribution of all unigenes. Each column in the figure shows one sample, each row represents one gene. The color in the figure indicates the size of the gene expression in the sample (log10 (FPKM+ 1)). Red indicates that the gene is highly expressed in the sample, and the blue indicates a lower expression level. The top of the tree is a cluster of samples, with the name of the sample below. **b** Cluster analysis by K-means method from gene expression profiles. The blue lines show expression model. The gray lines are all individual DEGs’ expression profiles. The x-axis represents the different tissues of *Dendrobium moniliforme*. The y-axis represents log_2_(ratio)

Based on the transcriptome data, the hierarchical clustering of DEGs (Fig. 4a) between different tissues indicated the abundance of DEGs by combining the FPKM with color. A total of 35,159 DEGs were identified and analyzed using criteria of log10 (FPKM+ 1) and *p* < 0.05. For the trend of the specific expression level, see the numbers under the color bar at the top left. The left is the tree of the gene cluster; the closer the two genes are separated, the closer they are expressed.

To reflect the major trends and tissue-specific expression between tissues in *D. moniliforme*, all DEGs were clustered into ten expression profiles (Fig. 4b) by the K-means method and hierarchical clustering with similar regulation model and log2 (foldchange). Unigenes belonging to cluster 4, 6 and 8 were more highly expressed in stems than in other tissues; unigenes belonging to cluster 3 and 5 were highly expressed in leaves. Interestingly, all unigenes mainly expressed in roots belong to a cluster of 2256 genes.

### Enrichment analysis and metabolic pathway assignment by KEGG

**Fig. 5:**
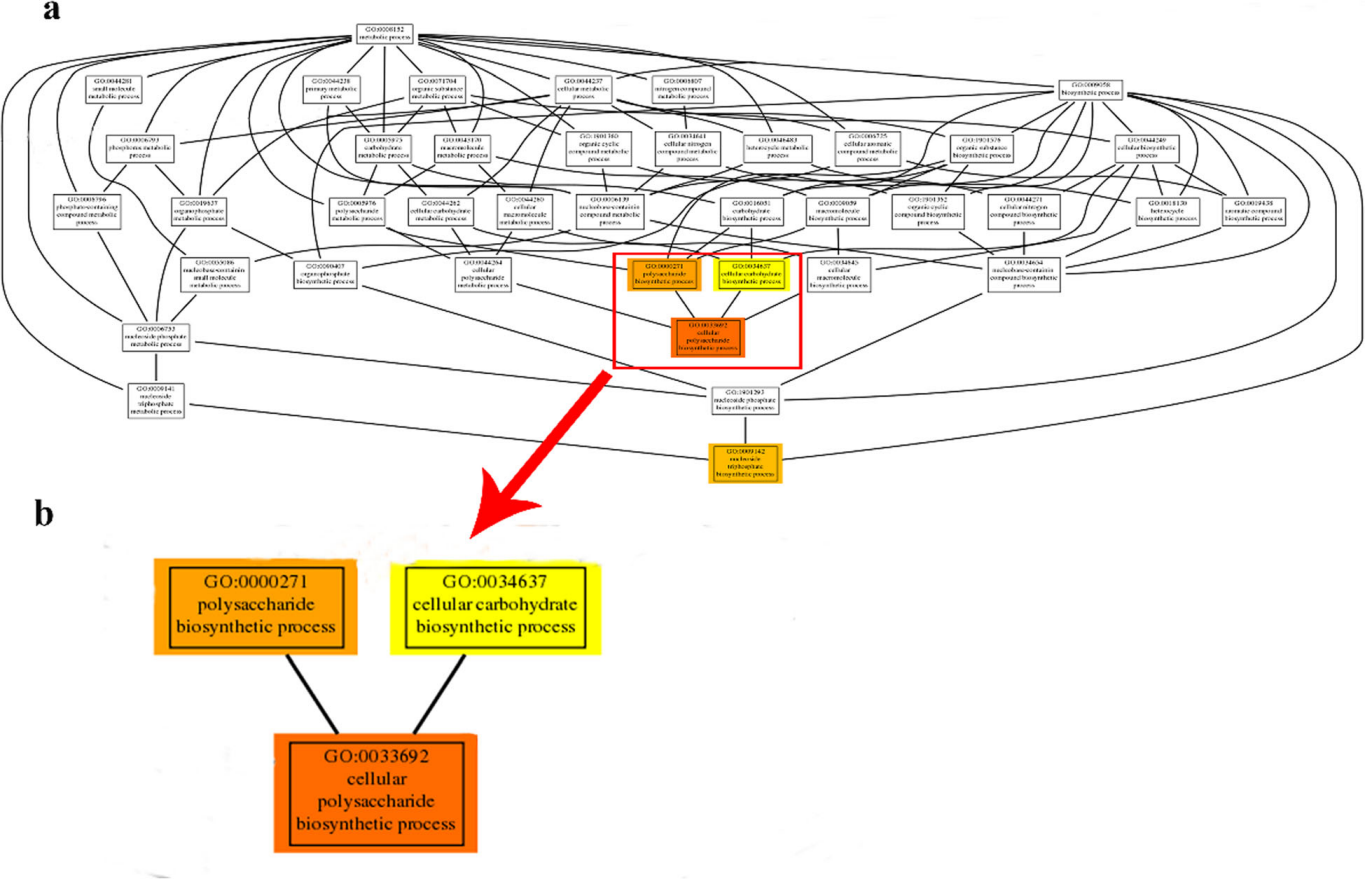
Thumbnails view of directed acyclic graph (DAG) on Molecular function between Dm_R vs. Dm_S. **a** Thumbnails view of DAGs on MF. The depth of the color represents the degree of enrichment, the deeper the color represents the higher the degree of enrichment. The node horizontal position means the depth of GO terms. **b** Information of three top enriched terms

Further, to gain a better understanding of the function clusters and biochemical pathways, we performed GO and KEGG enrichment analysis on all DEGs. In detail, 2188 GO terms were identified in “Dm_L vs. Dm_S” comparisons; 2691 GO terms were identified in “Dm_R vs. Dm_L” comparisons; while 1820 GO terms were identified in “Dm_R vs. Dm_S” comparisons. A figure for GO significant enrichment is shown in Additional file 1: Figure S3. By visualizing the GO term from GO enrichment analysis, we can see the correlation between these functions. The structure of GO can be described in the form of a directed acyclic graph (DAG) in which each GO term is a node and the parentage is an arrow. A thumbnail view of the directed acyclic graph (DAG) on molecular function between Dm_R vs. Dm_S is shown in Fig. 5. The top three GO terms, shown in expanded size, are polysaccharide biosynthesis process (GO:0000271), cellular polysaccharide biosynthesis process (GO:0033692) and cellular carbohydrate biosynthesis process (GO:0034637). Polysaccharides are the main medicinal composition of *D. moniliforme*, the result of GO enrichment is the same to it.

**Fig. 6:**
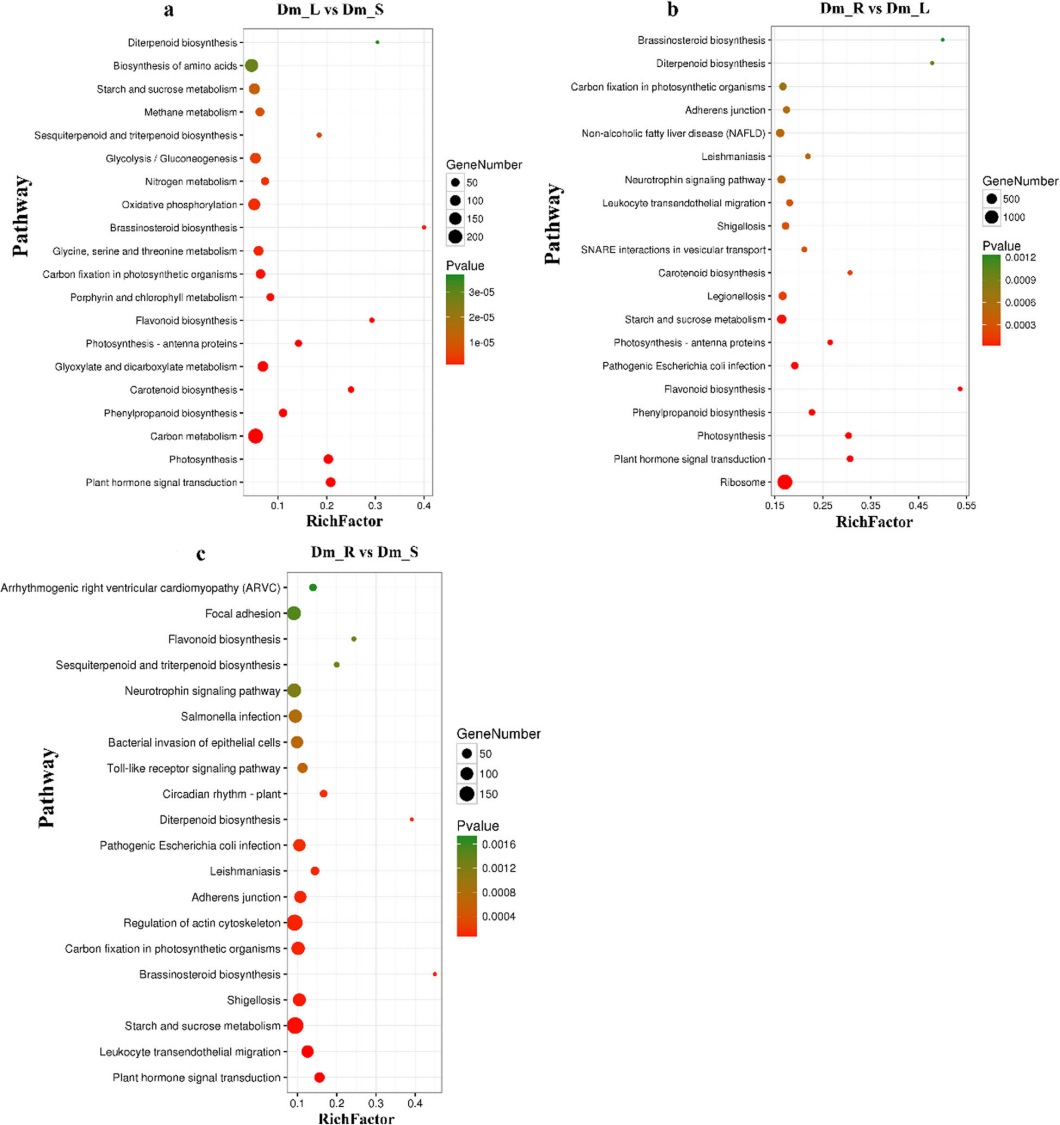
Top 20 of Kyoto Encyclopedia of Genes and Genomes (KEGG) pathway enrichment of DEGs. The x-axis indicates the pathway name, and the y-axis indicates the rich factor corresponding to the pathway. The q-value is represented by the color of the dot. The number of DEGs is represented by the size of the dots. **a** Dm_L vs. Dm_S; **b** Dm_R vs. Dm_L; **c** Dm_R vs. Dm_S

In the KEGG enrichment analysis (Additional file 1: Figure S4), KEGG pathway was divided into seven classes: Environmental Information Processing (EIP), Genetic Information Processing (GIP), Cellular Processes (CP), Organismal Systems (OS), Drug Development (DD), Human Diseases (HD) and Metabolism (M). In terms of the KEGG pathways, “Dm_L vs. Dm_S” comparisons are involved in 332 pathways with 8279 DEGs and 249,623 background unigenes. In “Dm_R vs. Dm_L” comparisons, 351 pathways were involved in and 32,085 DEGs and 250,769 background unigenes were identified. Finally, in “Dm_R vs. Dm_S” comparisons, 343 pathways were involved, and 17,090 DEGs and 250,453 background unigenes were identified. In general, when the corrected *p*-value < 0.05, the GO and KEGG enrichment is considered to be significant. The top 20 KEGG enrichment pathways are shown in Fig. 6.

Plant secondary metabolites play an important role in many aspects of plant life activities, and many plant secondary metabolites are necessary for plant life activities [29]. In this study, a total of 104,684 unigenes were assigned to 503 KEGG pathways. Among these pathways, 20 KEGG pathways related to secondary metabolites were identified, including anthocyanin biosynthesis (ko00942), betalain biosynthesis (ko00965), brassinosteroid biosynthesis (ko00905), flavonoid biosynthesis (ko00944), caffeine metabolism (ko00232), monobactam biosynthesis (ko00261) and others (Table 2). The phenylpropanoid biosynthesis (698 unigenes) pathway represented the largest unigenes group, followed by steroid biosynthesis (626 unigenes) and cyanoamino acid metabolism (600 unigenes). These pathways may provide resources for future study of the functions and processes during *D. moniliforme* development.

**Table 2:** The pathways and number of unigenes related to secondary metabolites pathway

Biosynthesis of secondary metabolic pathway	Pathway ID	Genes with pathway annotation (152,667)
Anthocyanin biosynthesis	ko00942	4 (0.002%)
Betalain biosynthesis	ko00965	98 (0.064%)
Brassinosteroid biosynthesis	ko00905	51 (0.033%)
Carotenoid biosynthesis	ko00906	168 (0.110%)
Caffeine metabolism	ko00232	92 (0.060%)
Cyanoamino acid metabolism	ko00460	600 (0.393%)
Diterpenoid biosynthesis	ko00904	76 (0.050%)
Flavone and flavonol biosynthesis	ko00944	22 (0.014%)
Flavonoid biosynthesis	ko00941	90 (0.059%)
Isoquinoline alkaloid biosynthesis	ko00950	385 (0.252%)
Monoterpenoid biosynthesis	ko00902	9 (0.006%)
Nicotinate and nicotinamide metabolism	ko00760	387 (0.254%)
Phenylpropanoid biosynthesis	ko00940	698 (0.457%)
Steroid biosynthesis	ko00100	626 (0.410%)
Terpenoid backbone biosynthesis	ko00900	598 (0.392%)
Tropane, piperidine and pyridine alkaloid biosynthesis	ko00960	292 (0.191%)
Monobactam biosynthesis	ko00261	168 (0.110%)
Streptomycin biosynthesis	ko00521	319 (0.209%)
Novobiocin biosynthesis	ko00401	64 (0.042%)
Aflatoxin biosynthesis	ko00254	104 (0.068%)

### Detection of candidate genes related to glycosyltransferases and cellulose synthase in *D. moniliforme*

**Fig. 7:**
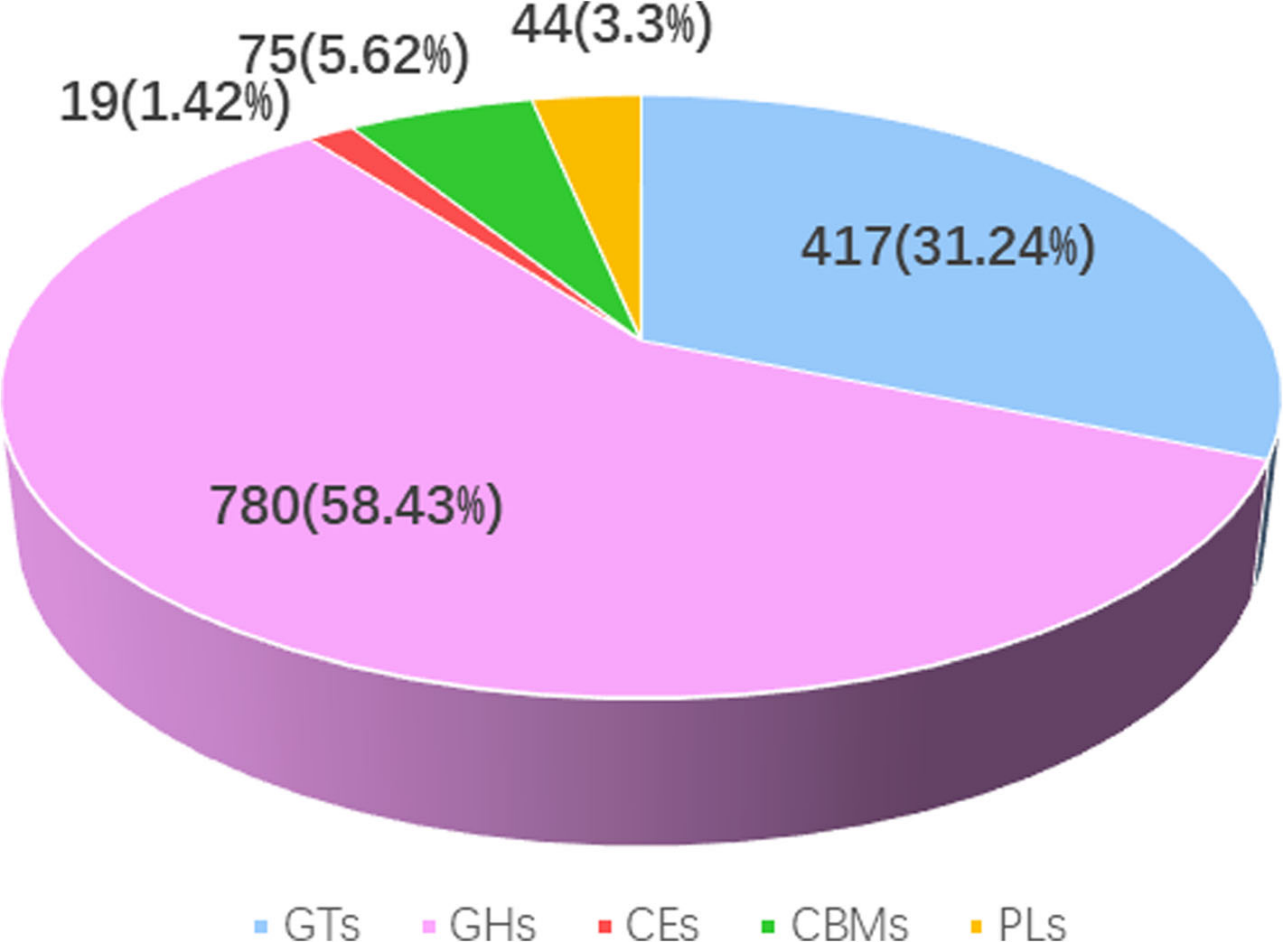
The classification and number of carbohydrate-active enzyme families in *D.moniliforme* unigenes. GT, Glycosyltransferase; GH, Glycoside Hydrolase; CE, Carbohydrate Esterase; CBM, Carbohydrate-Binding Module; PL, Polysaccharide Lyase

The carbohydrate-active enzyme database (CAZy) is a database resource for enzymes that can synthesize or disassemble complex carbohydrates and glycoconjugates. The carbohydrate active enzymes by functional classification include glycosyltransferase genes (GTs), glycoside hydrolases (GHs), carbohydrate esterases (CEs), carbohydrate-binding modules (CBMs) and polysaccharide lyases (PLs). We identified 1204 carbohydrate-active related unigenes against CAZy database using BLASTX (E < 0.00001), which include 417 GTs, 780 GHs, 19 CEs, 75 CBMs and 44 PLs (Fig. 7).

**Fig. 8:**
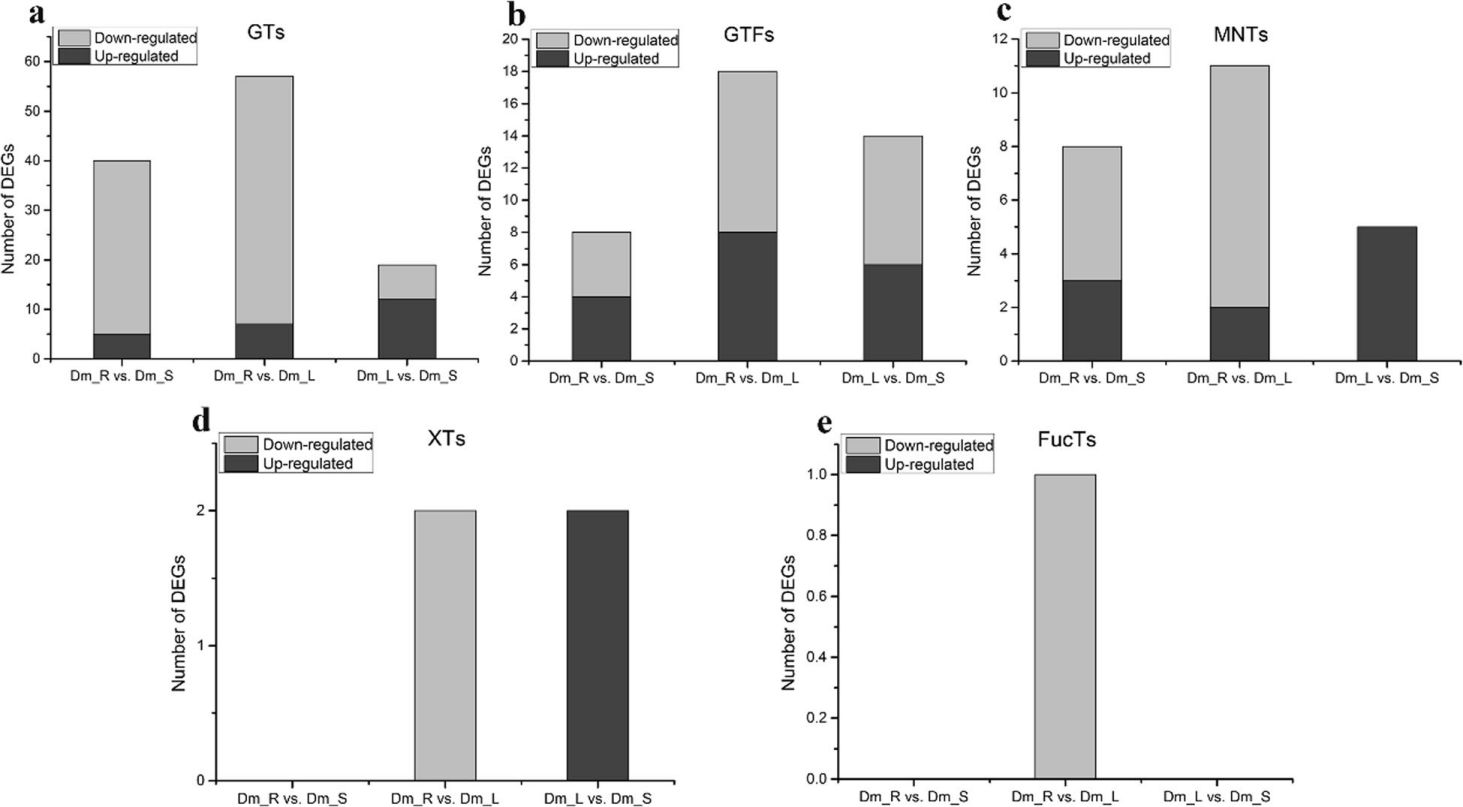
Analysis of differentially expressed (DEGs) related to polysaccharide. The number of up-regulated and down-regulated between Dm_L vs. Dm_S, Dm_R vs. Dm_L, Dm_R vs. Dm_S are summarized. **a** Glycosyltransferases; **b** Glucosyltransferases; **c** Mannosyltransferases; **d** Xylosyltransferases; **e** Fucosyltransferases

Glycosyltransferase is found in almost all organisms and catalyze the transfer of glycosyl groups, which is one of the most important for biotransformation and is directly involved in the biosynthesis of disaccharides, monosaccharides, oligosaccharides, alkyl glucosides and polysaccharides [30]. Among 417 glycosyltransferase genes (GTs), we identified several DEGs of GTs that exist in these three comparisons (Table 3). GTs account for a large proportion of the carbohydrate-related family. In the Dm_R vs. Dm_L comparison, 57 DEGs were identified including 7 up-regulated and 50 down-regulated of GTs. In fucosyltransferases (FucTs) of DEGs, only one down-regulated DEG was identified between the Dm_R vs. Dm_L comparison and no DEGs were found in the other two comparisons. In xylosyltransferases (XTs) of DEGs, a total of two up-regulated DEGs were identified between Dm_L vs. Dm_S and two down-regulated DEGs were found between Dm_R vs. Dm_L comparison (Fig. 8).

**Table 3:** The category and number of GT families in the DEGs database

Family	Number of DEGs (Dm_L vs. Dm_S)	Number of DEGs (Dm_R vs. Dm_L)	Number of DEGs (Dm_R vs. Dm_S)
GT1	12	24	15
GT2	6	15	9
GT5	1	0	1
GT15	0	1	1
GT20	0	4	4
GT22	0	2	2
GT31	0	1	1
GT34	0	1	1
GT35	0	2	2
GT41	0	1	1
GT48	0	4	3
GT62	0	1	0
GT66	0	1	0
Total	19	57	40

**Fig. 9:**
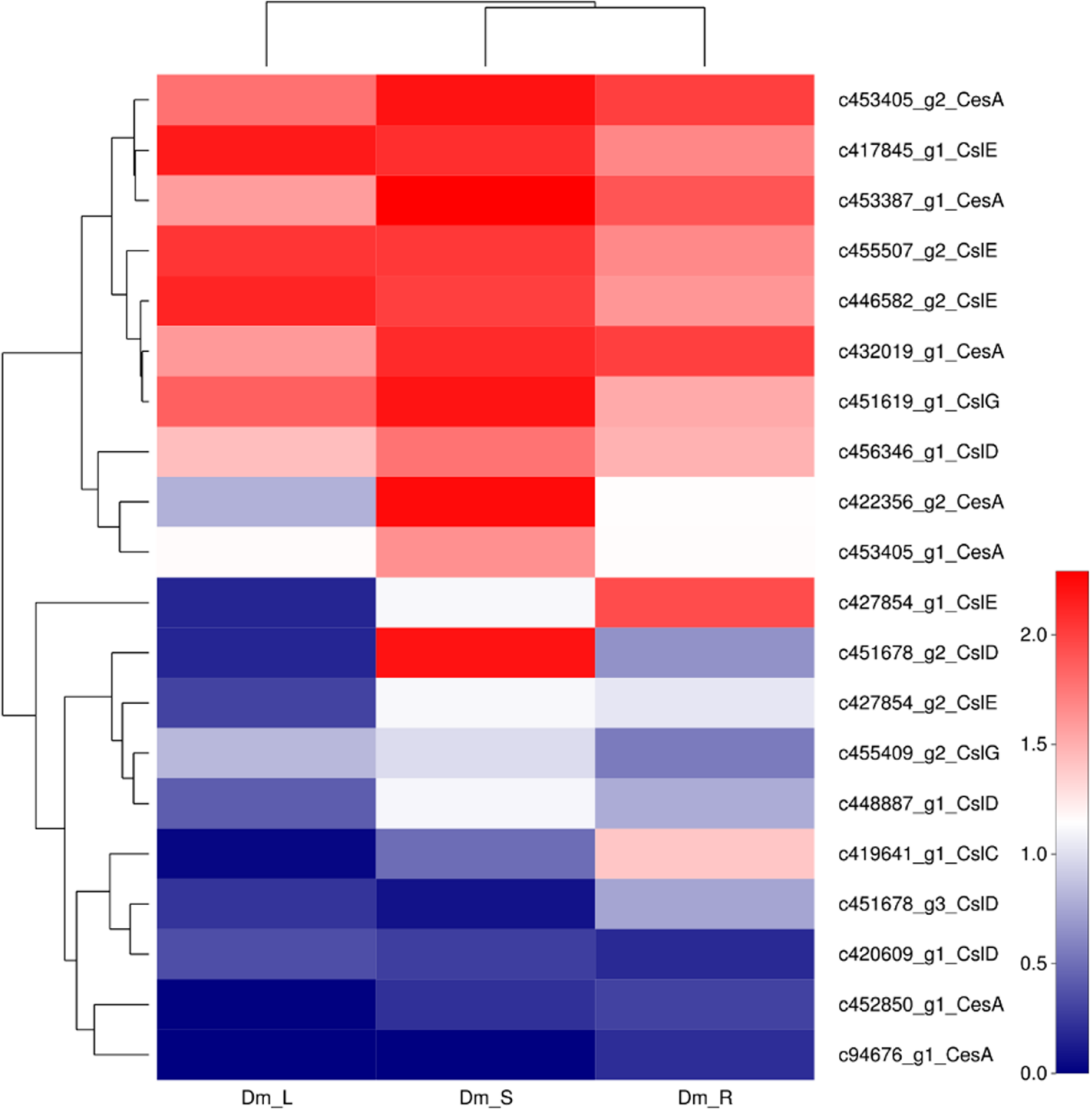
The heatmap of DEGs involved in cellulose synthase superfamily. Red indicates high expression genes, while green indicates low expression genes. Color from red to green indicate that log10 (FPKM+ 1) gradually changes from big to small

Cellulose is the most abundant organic matter in the biosphere and also is the structural polysaccharide of plants, the major component of their cell walls. The *D. moniliforme* cellulose synthase superfamily is divided into one CesA (cellulose synthase) family and nine Csl (cellulose synthase-like) families. Genes related to the cellulose synthase superfamily were involved in the synthesis of mannan polysaccharides (Liepman et al., 2005). We identified 35 putative unigenes for CesA in *D. moniliforme*, which were classified into six families, CesA, CslC, CslD, CslE, CslG, CslH. Among 35 CesA-related genes, 21 genes showed differential expression in Fig. 9. These CesA-related DEGs were divided into five families, CesA, CslC, CslD, CslE and CslG with 8 and 5 DEGs belonging to CesA and CslD families, respectively.

### Analysis of putative genes and pathways involved in DMP biosynthesis

Among 128 metabolic pathways detected in *D. moniliforme*, candidate genes related to starch and sucrose metabolic pathways were ranked in the top 20 as far as the number of involved genes was concerned (Table 4). To understand the biosynthesis of *D. moniliforme* polysaccharides (DMPs), the unigenes involved in the biosynthesis of starch and other related polymers, as well as those related to carbon assimilation in the tissues of stem, root and leaf, were annotated. Based on the KEGG database, we determined the key enzyme genes involved in these pathways (Table 3). The largest number of unigenes (17 unigenes) were identified as uridine-diphosphate glucose pyrophosphorylase (galU) encoding genes; and the second largest number of unigenes (14) were annotated as hexokinase (HK) encoding genes; while the third largest number of unigenes (11) were annotated as phosphoglucomutase (pgm) encoding genes.

**Table 4:** The number of DEGs involved in the biosynthesis of starch and sucrose in *Dendrobium moniliforme*

Enzyme Code	Enzyme Name	Abbreviation	Number of DEGs (Dm_L vs. Dm_S)	Number of DEGs (Dm_R vs. Dm_L)	Number of DEGs (Dm_R vs. Dm_S)
3.2.1.26	β-fructofuranosidase	sacA	1	2	2
5.4.2.2	Phosphoglucomutase	pgm	3	8	7
2.7.7.9	Uridine-diphosphate glucose pyrophosphorylase	galU	4	15	10
5.1.3.2	UDP-glucose 4-epimerase	GALE	3	8	4
5.1.3.6	UDP-glucuronate 4-epimerase	UGE	3	2	1
5.1.3.5	UDP-arabinose 4-epimerase	UXE	2	1	0
5.3.1.9	Glucose-6-phosphate isomerase	GPI	2	9	6
2.7.1.1	Hexokinase	HK	3	14	3
2.7.1.4	Fructokinase	scrK	1	0	0
5.3.1.8	Mannose-6-phosphate isomerase	MPI	3	3	3
5.4.2.8	Phosphomannomutase	PMM	1	2	2
2.7.7.13	Mannose-1-phosphate guanylyltransferase	GMPP	4	6	6
4.2.1.47	GDP-mannose 4,6-dehydratase	GMDS	1	4	2

**Fig. 10:**
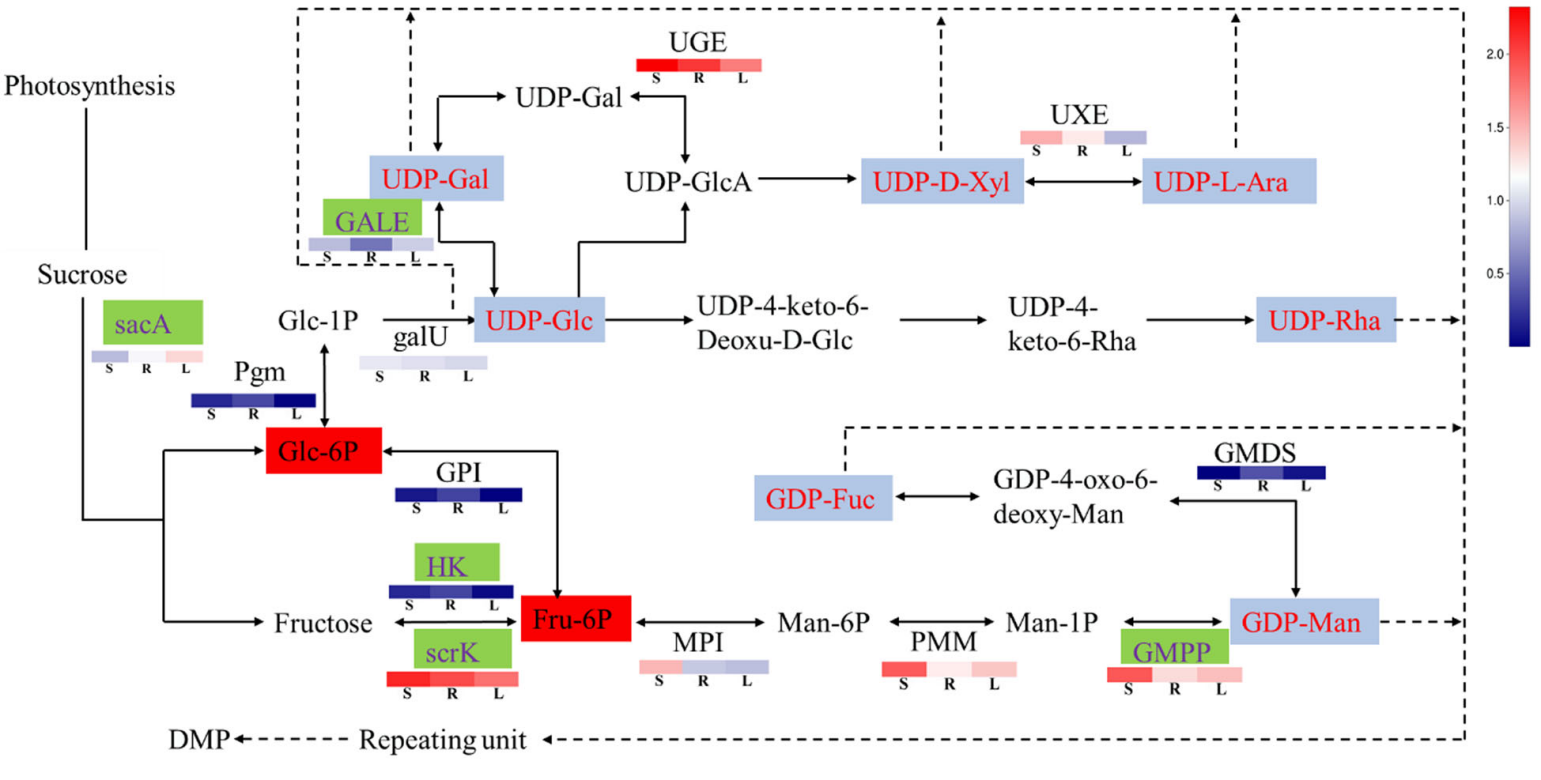
Proposed pathways for polysaccharide biosynthesis in *Dendrobium moniliforme*. Activated monosaccharide units, marked in red with purple background; key enzymes, marked in purple with green background. Bolded text with red background indicates key intermediates, various blocks represent the logarithms of FPKM values for different samples (stem, root and leaf from left to right). The real line arrows represent the identified enzymatic reactions, and the dashed line arrows represent multiple enzymatic reactions by multiple steps

In plants, UDP-glucose pyrophosphorylase (UGPase) catalyzes the formation of uridine diphosphate glucose (UDP-Glu), a key precursor of nucleotide-diphospho-sugar (NDP-sugar) formed from Glucose-1-phosphate (Glc-1P). Plant polysaccharides are formed by the active NDP-sugar precursors, which are added to the residues of polysaccharides and glycoconjugates by the action of various glycosyltransferases (GTs) [31, 32]. The composition of cell walls is determined in part by the availability of NDP-sugars to form different types of wall polymers [33]. Since all polysaccharides are synthesized from activated NDP-sugars, the biosynthetic pathway of DMPs was inferred and predicted (Fig. 10).

Previous studies indicated that DMPs are formed by the polymerization of NDP-sugars such as UDP-galactose (UDP-Gal), UDP-D-xylose (UDP-D-Xyl), UDP-L-arabinose (UDP-L-Ara), UDP-Glucose (UDP-Glc), UDP-rhamnose (UDP-Rha), GDP-mannose (GDP-Man) and GDP-fucose (GDP-Fuc) [34, 35]. DMP biosynthesis can be divided into three main stages. First, sucrose is converted to Glc-6P and Fru-6P, leading to the formation of UDP-Glc and GDP-Man from Glc-6P and Fru-6P, which are then converted in the second step into other NDP-sugars. Many enzymes play important roles in these processes, such as sacA [36], which converts sucrose to Glc-6P and Fructose, and pgm that isomerizes Glc-6P to Glc-1P [37]. HK [38] and scrK [39] also take part in the biosynthesis of Fru-6P. Secondly, UDP-Glc is derived from Glc-1P immediately [40], and Fru-6P is converted to GDP-Man indirectly [41]. Based on UDP-Glc and GDP-Man, other NDP sugars are further converted through the action of NDP-sugar interconversion enzymes (NSEs) [42], such as GALE, UGE, UXE and GMDS. Finally, the active monosaccharide units, NDP-sugars, are added to the sugar residues of various polysaccharides and glycoconjugates by the action of various GTs.

### Transcription factors involved in *D. moniliforme* transcriptome dataset

TFs have been implicated in a variety of developmental and physiological roles in plants. By comparison with the TFs from the iTAK database (http://itak.feilab.net/cgi-bin/itak/index.cgi). These TFs belong to 66 known TF families (Table 5), the most abundant being the C2H2 family,including 1158 unigenes. All these TFs have been identified as positive or negative regulators in the biosynthesis of secondary metabolites in other plants [43]. Previous genetic and molecular studies have revealed that NAC and MYB families, control secondary cell wall thickening in fibres, vessels, and anthers [44, 45] and control cellulose biosynthesis and assembling in cell walls [46]. We identified 133 MYB TFs and 90 NAC TFs in our transcriptome. MYB75 forms functional complexes to regulate secondary cell wall deposition and to integrate the metabolic flux through the lignin, flavonoid, and polysaccharide pathways in *Arabidopsis* [47]. SUSIBA2, a WRKY TF participated in sugar signaling by binding to the sugar-responsive elements of the iso1 promoter [48]. In our study, we discovered 88 WRKY TFs in the dataset. Through differential expression analysis, it was found that there were 30 differentially expressed MYB in roots, stems and leaves, 15 in NAC and 10 in WRKY.

**Table 5:** Transcription factor families identified in the *D. moniliforme* transcriptome dataset

Putative transcription factor family	Number of gene expressed in transcriptome	Putative transcription factor family	Number of gene expressed in transcriptome
AP2/ERF-ERF	118	LIM	47
B3	35	LOB	24
bHLH	221	MADS-M-type	62
bZIP	450	MYB	133
C2C2-YABBY	25	MYB-related	323
C2C2-Dof	41	NAC	90
C2C2-GATA	140	NF-YB	32
C2H2	1158	NF-YC	51
C3H	476	RWP-RK	35
E2F-DP	25	Trihelix	32
FAR1	50	WRKY	88
GARP-G2-like	77	zf-HD	13
GRAS	55	zn-clus	696
HB-other	131	others	423
HSF	108		

### Real-time quantitative RT-PCR verification

**Fig. 11:**
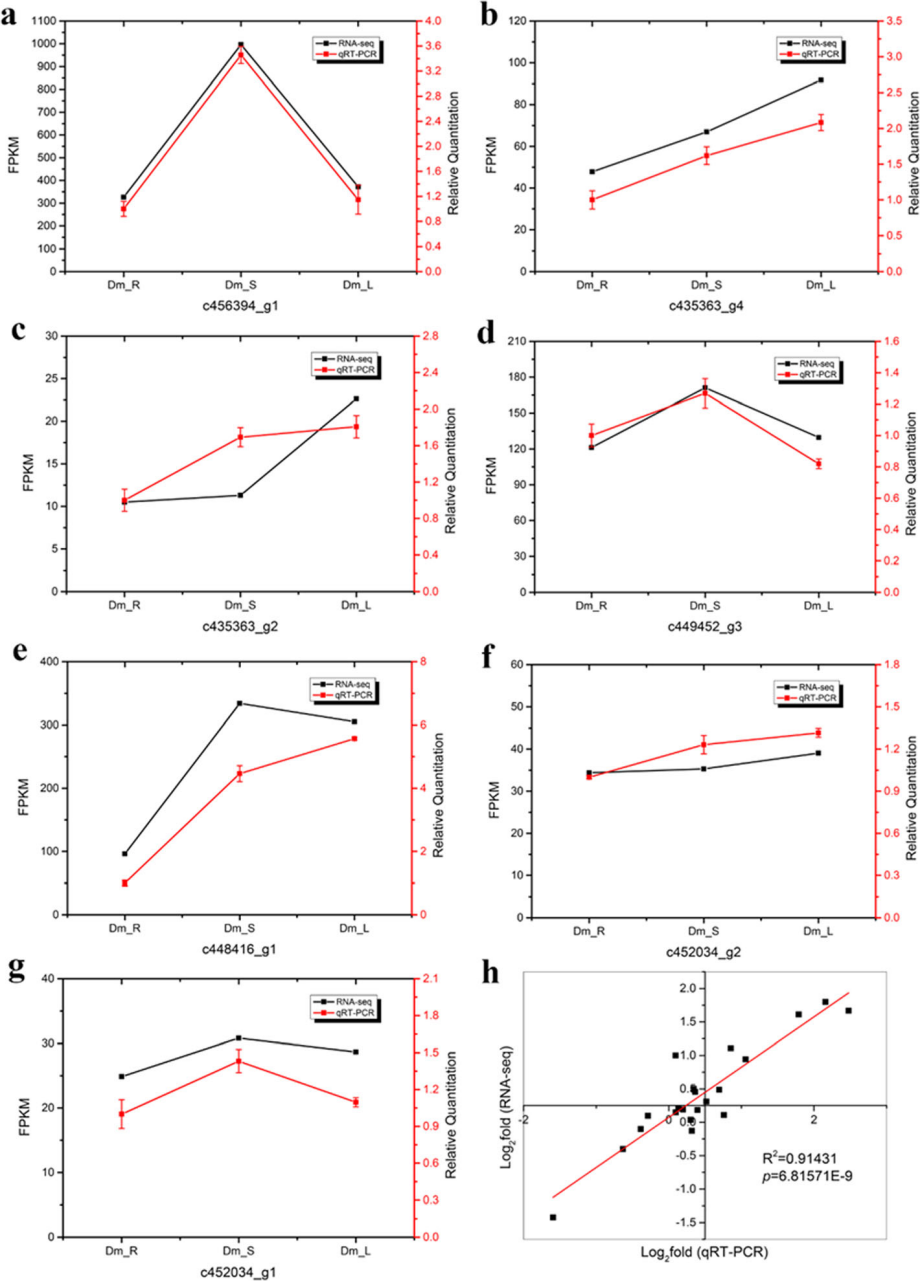
qRT-PCR verification compared with expression profiles of DEGs. The real-time RT-PCR results were highly in agreement with the RNA-seq data. The x-axis represents the tissues of root, stem and leaf. The y-axis represents log (foldchange). The title means unigene ID. (**a**-**g**) represent the expression patterns of two key enzyme-encoding genes: **a**-**d** Susy; **e**-**g** SPS. Correlation analysis is shown in (**h**)

To validate changes in gene expression patterns, we selected two key enzyme-encoding genes associated with polysaccharide biosynthesis, including sucrose synthase (Susy) (c456394_g1, c435363_g4, c435363_g2, c449452_g3) and sucrose phosphate synthase (SPS) (c448416_g1, c452034_g2, c452034_g1), and we examined these, using qRT-PCR at the transcriptional level. Primers and sequences are shown in Additional file 1: Table S1. The quantitative expression of DEGs is shown in Fig. 11a-g. Among all DEGs, the expression patterns of genes showed that the root samples had the lowest expression level of two key enzyme-encoding genes. However, we found that the two genes each accounted for half of the high expression in leaves and stems. The expression patterns of 2 DEGs were consistent with the transcriptome data (*R*^*2*^ = 0.91431, *p*-value = 6.81571E-9). These results indicate that our transcriptomic analysis was highly reproducible and reliable (Fig. 11h).

## Discussion

To date, this is the first time de novo sequencing of *D. moniliforme* has been conducted that has utilized the Illumina HiSeq platform. The sequencing produced 119.89 Gb clean data and 562,480 unigenes after assembly. The unigenes had the average distribution length of 703.77 bp, which is shorter than that from *D. officinale* (728 bp). We aligned to 169,702 unigenes (48.99%) by BLASTX in the Nr database, but in proximal species-*D. officinale*, 70,146 unigenes showed significant similarity to known proteins in the Nr database. Our annotated unigene numbers are almost 2.5 fold greater than the unigene numbers from a previous study that focused on three different tissues [28]. There were 37,245 unigenes assigned to KOG database for functional prediction and classification. Our annotated unigene numbers are 1.4 fold fewer than the unigene numbers in *D. officinale* [28]. This information provides more adequate resources to study *Dendrobium* species.

KEGG is a major public pathway-related database that is able to analyze a gene product during a metabolic process and related gene function incellular processes [49]. We annotated 104,684 unigenes in the KEGG database. They were assigned to 503 KEGG pathways; among them, 20 KEGG pathways related to secondary metabolites were identified in *D. moniliforme*. There are many secondary metabolites and their chemical structures are different. These secondary metabolites are mainly formed in the plant by the phenylpropanoid metabolic pathway, isoprene metabolic pathway and the alkaloid synthesis pathway [50]. The biosynthesis of phenylpropanoids occupies an important position in most plants, such as tea plants [51], grapes [52], *Polygonum minus* [53], and *D. moniliforme* is the same to them. This result is consistent with most other studies. However, in *Dendrobium*, polysaccharides are the most important active ingredients. In this study, we therefore focus on the synthesis of *D. moniliforme* polysaccharides.

Polysaccharides are the main active constituents of *D. moniliforme*, which have the positive effects on human health. Research on *D. moniliforme* polysaccharides has been of great interest, especially studies on their content, composition, and pharmacological effects [54]. However, there are few reports on polysaccharide biosynthesis pathways and genes of *Dendrobium* species. The biosynthesis of polysaccharides involves hundreds of different GTs. The latest CAZy (http://www.cazy.org/GlycosylTransferases.html) indicates that the GTs are classified into 105 families, of which 42 and 43 GT families belong to *Arabidopsis thaliana* and *Oryza sativa*, respectively, with a total of 463 and 574 GT genes. In this study, we identified 413 putative GTs in the *D. moniliforme* transcriptome database that were divided into 13 families. In Dm_L vs. Dm_S, there are 19 DEGs of glycosyltransferase genes, belonging to 3 families (GT1, GT2 and GT5). In Dm_R vs. Dm_L, the largest number of DEGs was 57, which belong to 12 families. Compared with the other two comparisons, Dm_R vs. Dm_S has an intermediate number of 40 DEGs of glycosyltransferase genes, belonging to 10 families (Table 4). As can be seen from the above results, there are significant differences in the number of genes differentially expressed in aerial parts (leaf and stem) and root of *D. moniliforme*, but the differences in leaf and stem of aboveground parts are not significant. The stem of *Dendrobium* is the medicinal part. The medicinal ingredients all accumulate in the aerial parts, while the roots, which absorb nutrients from the matrix, transfer the nutrients to the aerial parts, so the content of the medicinal ingredients accumulated in the roots is not high. The GT1 family has the largest number among all three comparisons. It is a major GT family in plants and is also known as UDP glycosyltransferase (UGT) [55]. The UGT family, has about 120 UGTs in *Arabidopsis*, including eight distinct pseudogenes [56]. The second group consists of the GT2, GT20 and GT48 families, each accounting for approximately 10% of the genes. Glycosyltransferases, a multigene family that is widely involved in multiple metabolic pathways, need to respond to various changes in plant growth, the developmental environment, and other biological processes. Therefore, subsequent research on plant glycosyltransferases will have important scientific significance.

The family of mannans is the most wide spread group of polysaccharides in higher plants [57]. Cellulose synthase (CesA) superfamily genes were involved in the biosynthesis of mannan polysaccharides [58]. Cellulose is a major component of primary and secondary walls of plants and is synthesized from a rose-like protein complex of the CesA subunit, which consists of six rose-like subunits forming a larger rosette protein complex [59]. CesAs belongs to the glycosyltransferase-2 (GT-2) superfamily, which is classified into one cellulose synthase (CesA) family and nine cellulose synthase-like (Csl) families. The Csl families were divided into CslA/B/C/D/E/F/G/H/J. In 1996, Pear et al. identified the first *CesA* gene in higher plants, *GhCesA*1 from *Gossypium hirsutum* [60]. The University of Michigan cloned *PtrCesA*1, the first cellulose synthase gene in tree wood from *Populus tremuloides*, a cellulose synthase gene specifically expressed in the xylem, and which is involved in the formation of secondary walls [61]. After that, 10 *AtCesA* from *Arabidopsis thaliana*, 18 *PtriCesA* from *Populus trichocarpa* and some *CesA* genes from *Populus tremuloides* and *Eucalyptus camaldulensis* were identified [62–64]. There are also many studies that have demonstrated that the CesA and CslA family are involved in the biosynthesis of mannan polysaccharides in many plant species. A β-mannan synthase (ManS) gene belonging to the CslA family from *Cyamopsis tetragonolobus*, creates the β-1, 4-mannan backbone of galactomannan [65]. The CslA proteins from *Arabidopsis* is capable of producing β-mannan polysaccharides [66]. The research of these genes has laid a solid foundation for the resolution of other plant cellulose synthesis mechanisms. We identified 35 putative unigenes for CesA in the *D. moniliforme* RNA-seq data, which were classified into six families, CesA, CslC, CslD, CslE, CslG, and CslH. CesA is expressed in all plant tissues and in different types of cells. However, the expression of different members of this gene family in the primary wall to secondary wall formation is different. Some studies have shown that the Csl families are involved in the biosynthesis of mannan polysaccharides. For instance, CslC subfamily members encode β-1,4-glucan synthase [67], CslD members have been implicated in cellulose synthesis [68]. while the CslG and CslH subfamily members are involved in the biosynthesis of β-(1,3;1,4)-D-glucan [69, 70]. Based on the transcriptome database, the expression of CslG and CslH in the stem is higher than in other tissues, which might encode some enzyme responsible for the synthesis of mannan polysaccharides in *D. moniliforme*.

Polysaccharide is the decisive factor in the quality of *D. moniliforme*. Sucrose also provides the substrate for the accumulation of polysaccharides. Therefore, we selected Sucrose Synthase (Susy) and Sucrose Phosphate Synthase (SPS), which are closely related to sucrose, for quantitative RT-PCR verification. SPS is one of the key enzymes that catalyze the synthesis of sucrose in the cytoplasm and are necessary for sucrose to enter various metabolic pathways. In a study of the tomato fruit, N’tchobo et al. considered SPS to play an important role in increasing tomato fruit sugar content and sucrose accumulation. When the SPS activity is higher, the soluble sugar content and sucrose content is higher [71]. Studies have shown that in sugarcane stems and leaves of sucrose content and SPS activity were positively correlated [72]. Most of Susy exists in the cytoplasm, which can catalyze the synthesis and decomposition of sucrose. It is speculated that SuSy activity directly affects the synthesis and metabolism of sucrose in plant organs and regulates the sucrose-starch metabolism of the organs [73, 74]. Tian et al. found that the accumulation of sucrose was mainly regulated by SuSy during the ripening of grape berries and strawberry fruits [75]. During the growth and development of grape berry, the expression level of SuSy is increased, which plays a leading role in the regulation of sugar metabolism in fruit [76]. In *D. moniliforme*, the expression of SPS and Susy in stems and leaves was significantly higher than that in roots, which were also affected by the regulation of SPS and Susy.

## Conclusions

*D. moniliforme* is a famous Chinese herbal medicine and mainly distributed in tropical and subtropical regions. Polysaccharides are the main medicinal ingredients. To elucidate the molecular mechanisms of polysaccharides in *D. moniliforme*, we collected three different tissues and subjected them to high-throughput sequencing. A total of 562,480 unigenes were obtained in nine transcriptome libraries of *D. moniliforme*. In addition, 417 glycosyltransferases and 35 cellulose synthase genes were identified. Comparative analysis of the transcriptome in different tissues of *D. moniliforme* revealed a total of 35,159 DEGs that were mainly correlated with metabolic pathways and the biosynthesis of secondary metabolites. Our results provide understanding of the biosynthesis of DMPs at the molecular level in *D. moniliforme*. As the first report on the high-throughput sequencing of *D. moniliforme*, this study should provide novel insights into polysaccharides-related genes for *D. moniliforme* and should be a valuable molecular basis for study of *Dendrobium* spp.

## Methods

### Plant materials

*D. moniliforme* plants were artificially cultivated in the greenhouse of Anhui Tongjisheng Biotechnology Company, Lu’an, China. The original source was collected by the company from the wild after obtaining local permission. Seed germination and growth of protocorm-like bodies were cultured on half-strength Murashige and Skoog (MS) medium adding 6-BA 0.1 mg·L^− 1^, NAA 0.5 mg·L^− 1^ and 1% additives (30 g·L^− 1^ sucrose + 4 g·L^− 1^ agar + 20% potato) under a 12/12 h light–dark cycle (approx. 30 μmol m^− 2^·S^− 1^) at 25 ± 2 °C. After the age of 6 months, the plants were transplanted into pots and placed in the greenhouse at a temperature of 25–27 °C with a light/dark cycle of 12/12 h and 60–70% relative humidity. Roots, stems and leaves of 2-year-old plants were harvested separately from three biological replicates in March, 2017. All of the *D. moniliforme* samples were frozen in liquid nitrogen and stored at − 80 °C in an ultra-low temperature freezer for further processing. The voucher specimens were authenticated by Professor Jinchi Zhang and deposited at Jiangsu Province Key Laboratory of Soil and Water Conservation and Ecological Restoration in Nanjing Forestry University, Nanjing, China (Voucher number: 17C003).

### Determination of polysaccharide content

Leaf, stem and root samples were collected from 2-year-old *D. moniliforme* at maturation stage. The phenol-sulfuric acid method was applied to determine the polysaccharide contents in different tissues. The polysaccharide content was determined by using a glucose standard. Polysaccharides were extracted according to the method described by Chinese Pharmacopoeia (version 2010). Initially, samples (0.3 g) were precisely weighed, 200 ml of water was added, and the samples heated and refluxed for 2 h, filtered, then diluted to 250 ml. We accurately measured 5 ml of the solution into a 50 ml centrifuge tube, added 25 ml of ethanol, shook and refrigerated this for 1 h, centrifuged at 4000 r·min^− 1^ for 20 min, discarded the supernatant, and centrifuged again with 20 ml of 80% ethanol (same as above). We repeated these steps 2 times, poured off the supernatant, and dissolved precipitate in heated water to a constant volume of 50 ml. 1 ml of sample solution was put in the test tube to which 1 ml of 5% phenol solution was added. The solution was mixed thoroughly and 5 ml of concentrated sulfuric acid was added, shaken and placed in a 75 °C water bath for 20 min. Afterwards, it was cooled until it was at room temperature, the absorbance was measured at 490 nm using UV-visible spectrophotometer with 1 ml of water as a blank, and the test was performed in parallel three times.

### RNA isolation, cDNA library construction and sequencing

Total RNAs were extracted from these materials using OmniPlant RNA Kit (Cwbio, China). The purity of total RNA was analyzed using a Nanodrop 2000 Spectrophotometer (IMPLEN, USA). Total RNA quantity and quality were evaluated using NanoDrop 2000 spectrophotometer Agilent 2100 Bioanalyzer (Agilent Technologies, USA) and by agarose gel electrophoresis. According to the manufacturer’s instructions, sequencing experiments were performed using the TruSeqTM RNA Sample Preparation Kit (Illumina, USA). In short, the protocol consists of the following steps: beads with oligo (dT) were used to isolate poly(A) mRNA from total RNA and cut into short fragments of 300 bp by adding fragmentation buffer. Taking these short fragments as templates, random hexamer primer was used to synthesize the first-strand cDNA, and then the second-strand cDNA was synthesized to form a stable double-stranded structure. The products were purified and enriched by PCR to create the final cDNA libraries. Finally, the library sequencing was performed on Illumina Hiseq 4000 platform (Illumina Inc., USA).

### De novo transcriptome assembly and unigenes annotation

To get high-quality clean reads, we removed adapter-containing, low-quality reads and poly-N from the raw data by using SeqPrep (https://github.com/jstjohn/SeqPrep) and Sickle (https://github.com/najoshi/sickle). Meanwhile, the calculation of Q20, Q30, GC-content and sequence duplication level were calculated based on the clean reads. All clean reads were assembled using Trinity software [77] based on the left.fq and right.fq, with the min_kmer_cov set as 2 by default and all other parameters set as their default.

Functional annotation of all the assembled unigenes was done by performing homology search against five major public databases. All the unigenes were annotated using BLASTx, with a cut-off E-value of 10^− 5^ was taken as the threshold for significance. Five databases are as follows: Nr (NCBI non-redundant protein sequences, http://www.ncbi.nlm.nih.gov), Pfam (Protein family, http://pfam.sanger.ac.uk/), String (Search tool for the retrieval of interacting genes, http://www.string-db.org/), Swiss-Prot (A manually annotated and reviewed protein sequence database, http:// www.uniprot.org) and KEGG (Kyoto encyclopedia of genes and genomes, http://www.genome. jp/kegg/).

### Identification of differentially expressed genes (DEGs)

Gene expression level of each sample was estimated by RSEM version 1.2.15 [78]. The R Bioconductor package, edgeR [79] was used to identify the differentially expressed genes (DEGs) of two samples. The false discovery rate (FDR) criterion was used to calculate the threshold *P*-value in significance tests. To judge the significance of gene expression differences, we used FDR < 0.05 and |log_2_ (fold change)| ≥1 as the threshold. GO enrichment analysis of DEGs was implemented by the Goatools [80]. For pathway enrichment analysis, KEGG enrichment analysis was performed using KOBAS version 2.0.12 [81].

### Quantitative real-time PCR (qRT-PCR)

Studies have demonstrated that sucrose phosphate synthase (SPS) and sucrose synthase (Susy) are involved in sucrose metabolism [27]. These two genes were analyzed using qRT-PCR. Total RNA was extracted, as indicated above using new plant material, and three biological replicates were made. The specific primers for DEGs were designed by Oligo 7 software. The qRT-PCR was performed on an ABI 7500 Real-time PCR system (Applied Biosystems, USA) using SYBR Premix Ex Taq (Takara, Japan) according to the manufacturer’s instructions. The expressions of SPS and Susy were normalized against Actin [25], an internal reference gene. All gene IDs and the primer sequences are listed in the Additional file 1: Table S1.

## Supplementary information

**Additional file 1: Figure S1**: Sequence-length distribution of transcripts and unigenes assembled from Illumina. **Figure S2.** Venn diagram of all unigenes with annotations against five public databases. **Figure S3.** Functional gene ontology classification of unigenes. **Figure S4.** Kyoto Encyclopedia of Genes and Genomes (KEGG) pathway enrichment of DEGs.**Table S1**. Genes IDs and primers used in the quantitative real-time PCR (qRT-PCR) experiments.

**Additional file 2.** Differential expression genes between Dm_L and Dm_S.

**Additional file 3.** Differential expression genes between Dm_R and Dm_L.

**Additional file 4.** Differential expression genes between Dm_R and Dm_S.

## Data Availability

All sequence data have been deposited at the National Center for Biotechnology Information short read archive (SRA) under the accession number SRP139000.

## Abbreviations

CAZy: The carbohydrate-active enzyme
CBMs: Carbohydrate-binding modules
CEs: Carbohydrate esterases
CesA: Cellulose synthase
CesA: Cellulose synthase genes
Csl: Cellulose synthase-like.
DAG: Directed acyclic graph
DEGs: Differential expression genes
DMP: *Dendrobium moniliforme* polysaccharide
FDR: False discovery rate
FucTs: Fucosyltransferases
GHs: Glycoside hydrolases
GTs: Glycosyltransferase genes
KEGG: Kyoto encyclopedia of genes and genomes
Nr: NCBI non-redundant protein sequences
Pfam: Protein family
PLs: Polysaccharide lyases
SPS: Sucrose phosphate synthase
String: Search tool for the retrieval of interacting genes
Susy: Sucrose synthase
XTs: Xylosyltransferases
