# Supplementary material for: Polysaccharide biosynthetic pathway profiling and putative gene mining of Dendrobium moniliforme using RNA-Seq in different tissues
Source: BMC Plant Biol. 2019 Nov 27;19:521. doi: 10.1186/s12870-019-2138-7 (PMC6882186; doi:10.1186/s12870-019-2138-7)
Supplement: Supplementary file 1 — Additional file 1: Figure S1: Sequence-length distribution of transcripts and unigenes assembled from Illumina. Figure S2. Venn diagram of all unigenes with annotations against five public databases. Figure S3. Functional gene ontology classification of unigenes. Figure S4. Kyoto Encyclopedia of Genes and Genomes (KEGG) pathway enrichment of DEGs.Table S1. Genes IDs and primers used in the quantitative real-time PCR (qRT-PCR) experiments. [file 12870_2019_2138_MOESM1_ESM.docx]

**Supplementary File**


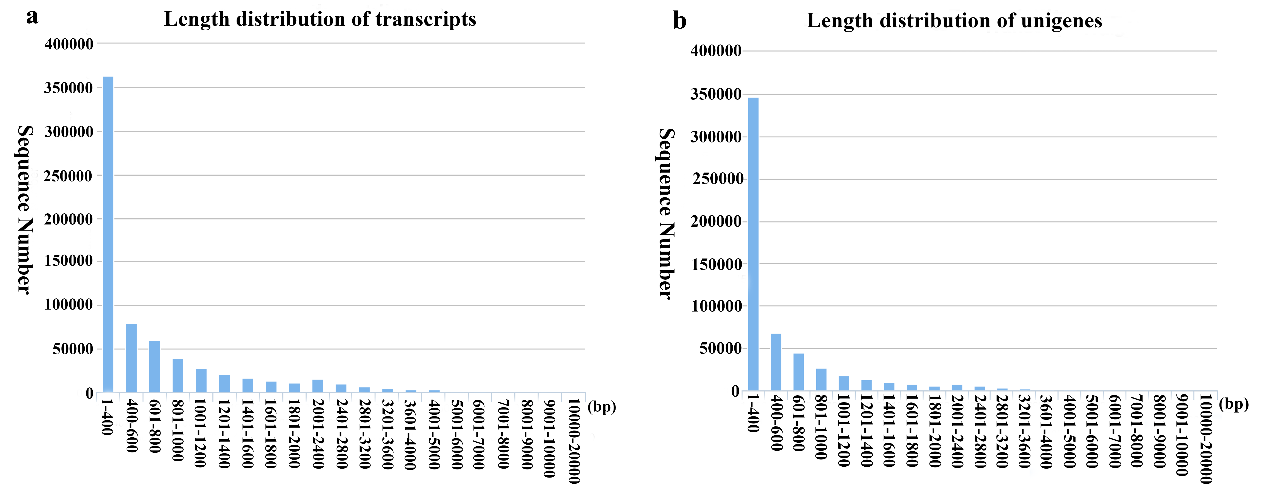


**Figure S1 Sequence-length distribution of transcripts and unigenes assembled from Illumina reads.** (a) Length distribution of transcripts; (b) Length distribution of unigenes.


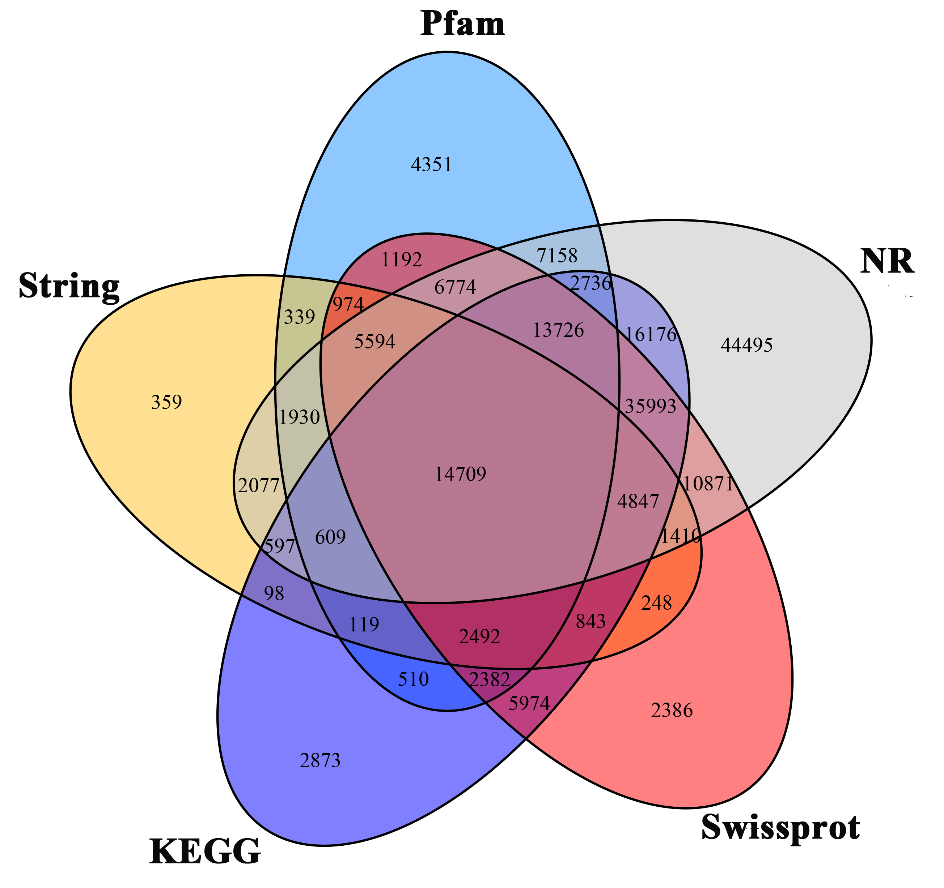


**Figure S2 Venn diagram of all unigenes with annotations against five public databases.**


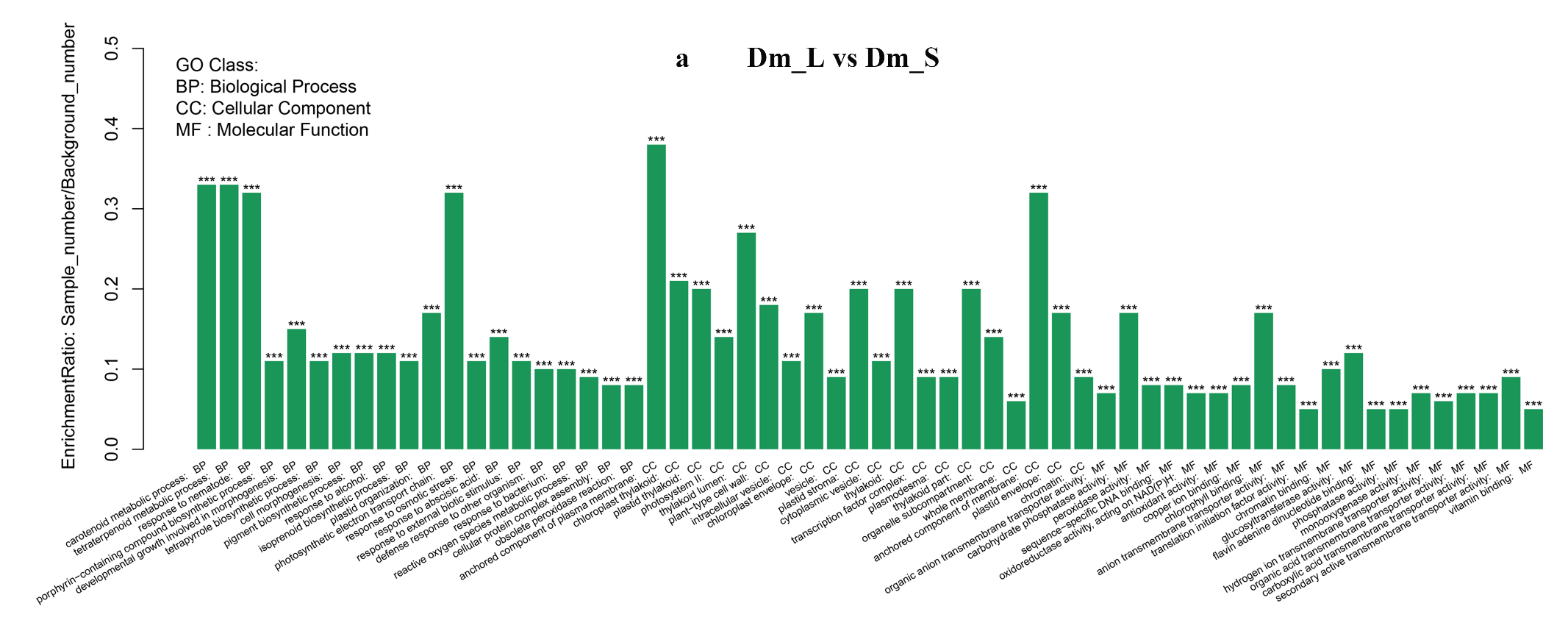


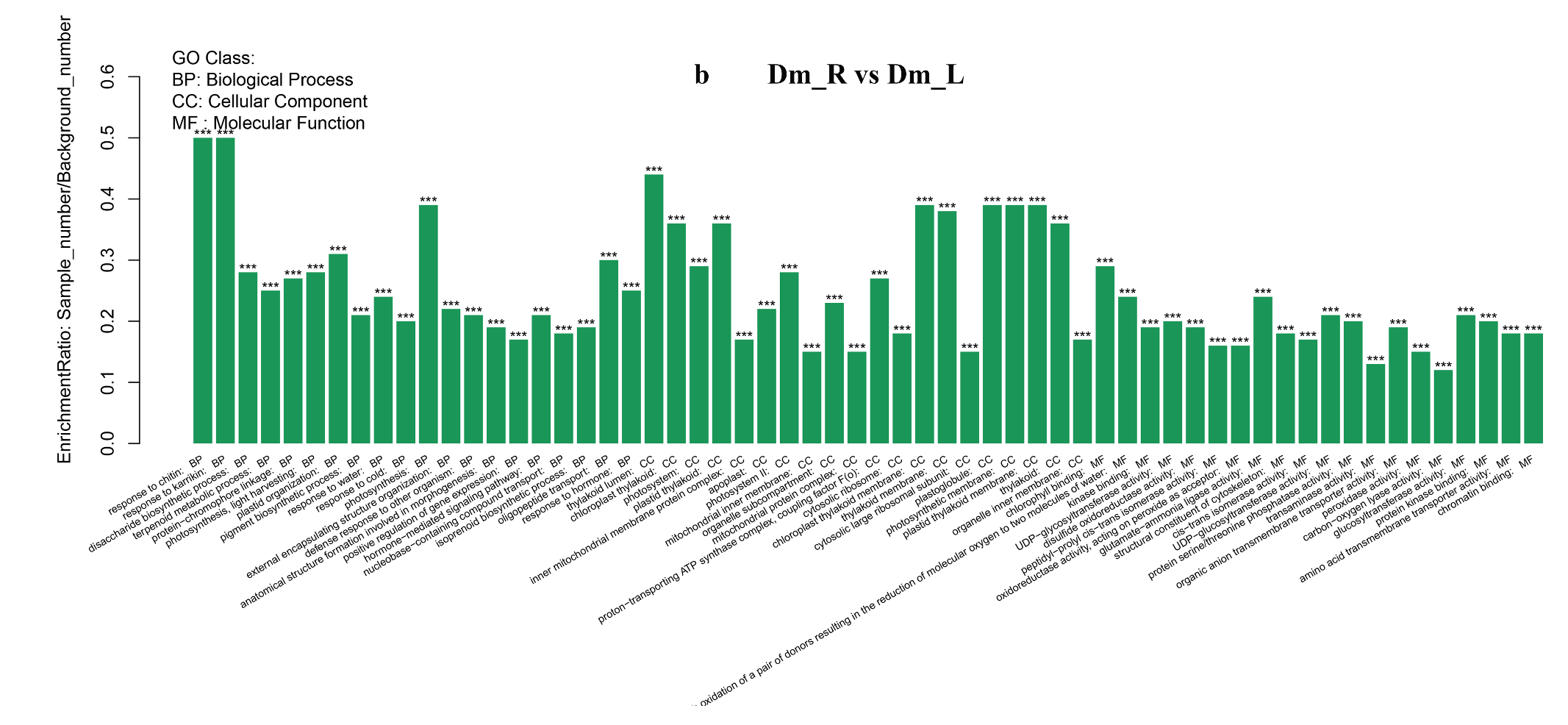


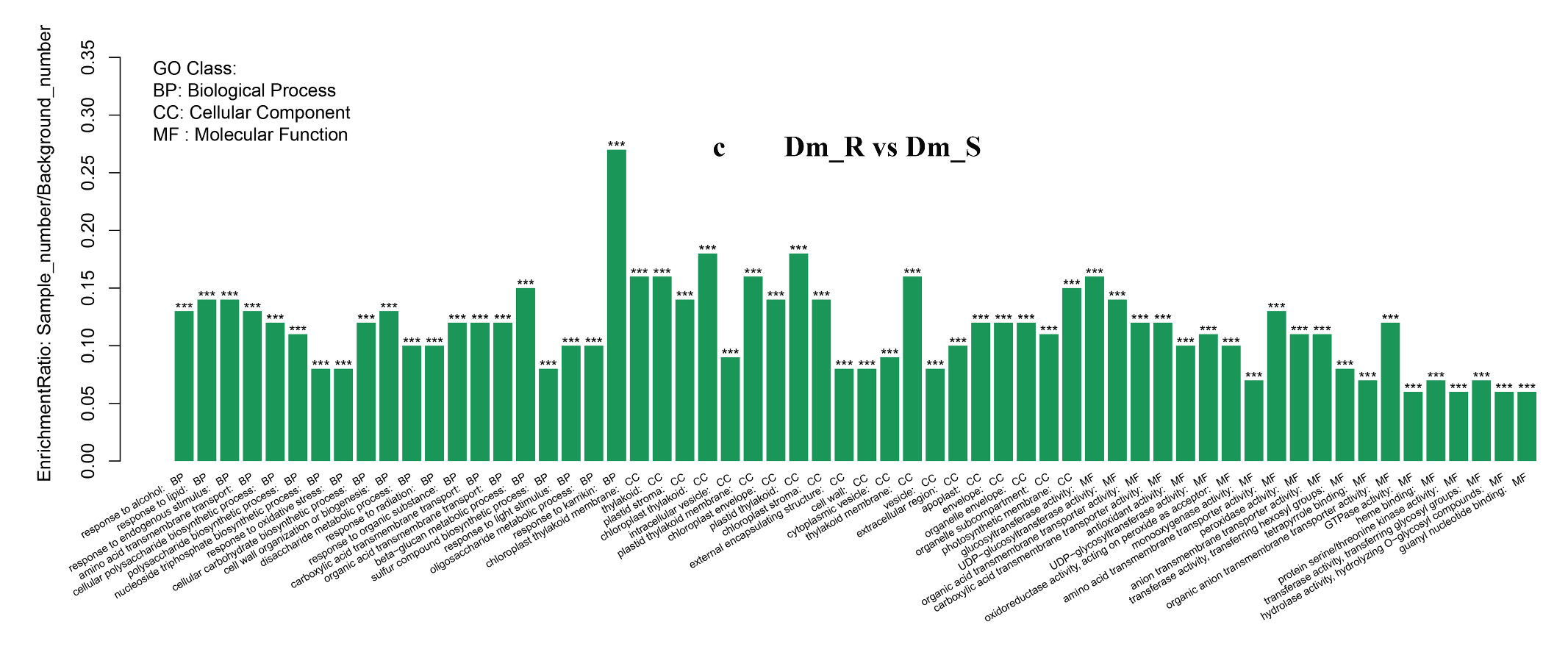


**Figure S3 Functional gene ontology (GO) enrichment of DEGs.** The y-axis indicates the enrichment ratio of unigenes in a category. (a) Dm_L vs. Dm_S; (b) Dm_R vs. Dm_L; (c) Dm_R vs. Dm_S. All pathways in the figure indicate significant GO enrichment, with three asterisks indicating FDR＜0.001.


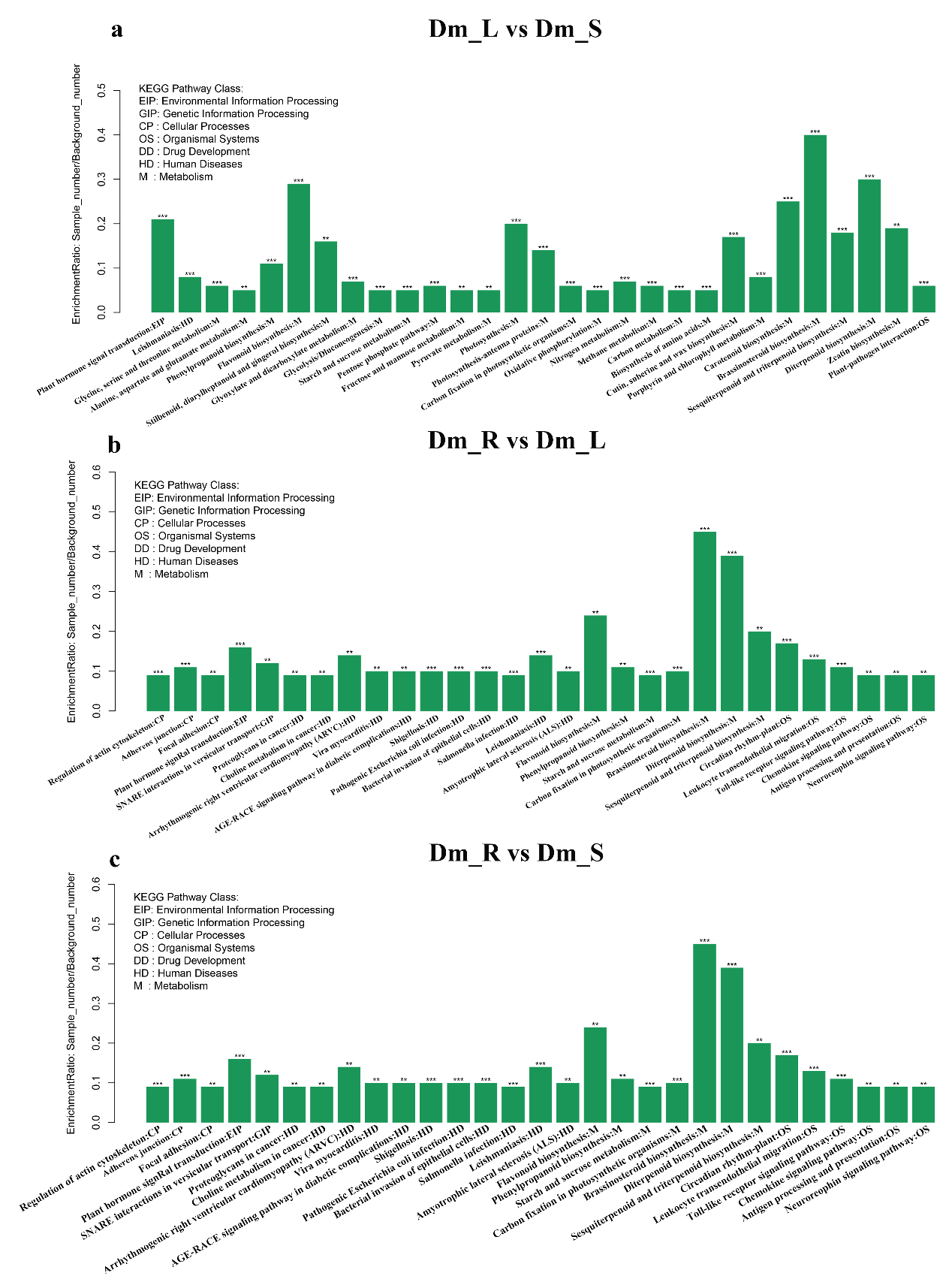


**Figure S4 Kyoto Encyclopedia of Genes and Genomes (KEGG) pathway enrichment of DEGs.** The y-axis indicates the enrichment ratio of unigenes in a category. (a) Dm_L vs. Dm_S; (b) Dm_R vs. Dm_L; (c) Dm_R vs. Dm_S. All pathways in the figure indicate significant KEGG enrichment, with three asterisks indicating p-value＜0.001 and two asterisks indicating p-value＜0.01.

**Table S1 Genes IDs and primers used in the quantitative real-time PCR (qRT-PCR) experiments.**

| Primer_ID | Forward PCR Primer (5′-3′) | Reverse PCR Primer (5′-3′) |
| --- | --- | --- |
| *Actin* | GGGAGCCACCACCTTGATCTTC | GTTTGCTGGCATTGCTGACAGG |
| c448416_g1 (*SPS*) | CCATTGCTGATGCGCTGCTG | GCGGCGACACGAGAAAGGTA |
| c452034_g2 (*SPS*) | GCCCATCTCACGTGCAAGTACC | TGCGGATTCAGGCACTTCGT |
| c452034_g1 (*SPS*) | CGACGAGGAGTCCGCAAGAG | GGCGGCCTCTTTCACGTTCT |
| c456394_g1 (*Susy*) | CACTGGCCATCCGACCAAGG | CACGCTCAGCTCCTCCACAG |
| c435363_g4 (*Susy*) | CCGCGTAGTTCACGGGATCG | TTCAGGGTGGAGGGCTGTGA |
| c435363_g2 (*Susy*) | GCAAGTACTCGGGCACAGTCA | GGGTTGCTCTTGCGATCCGA |
| c449452_g3 (*Susy*) | CCATGGCGACGCAGAAGCTA | CAAGGAGGTCATGCGGCTGT |
